# A quasi-two-stage heat pump for efficient dual—temperature supply—space heating and domestic hot water

**DOI:** 10.1038/s44172-026-00739-9

**Published:** 2026-07-21

**Authors:** Zahra Hajabdollahi Ouderji, Muhammad Abid, Zhibin Yu

**Affiliations:** Department of Mechanical and Aerospace Engineering, University of Liverpool, Liverpool, UK

**Keywords:** Mechanical engineering, Energy infrastructure, Energy harvesting

## Abstract

Heat pumps play a critical role in delivering space heating and domestic hot water in residential buildings. However, managing these two demands efficiently remains a technical challenge, especially when high water temperatures are required. This study introduces a dual-mode flexible heat pump operating in two distinct modes: during charging mode, it provides space heating while recovering heat from the refrigerant into an integrated latent thermal energy storage unit. During discharging mode, this stored energy supplies domestic hot water at higher temperatures. A performance analysis was carried out using refrigerants under different domestic hot water demand levels and space heating temperatures. Propane achieved a power reduction of 13.2% compared to a standard system at a 22% domestic hot water share. Further analysis showed that R1234yf delivered the highest power reduction of 14.3%, followed by R1234ze, propane, and isobutane, all within 13.1–13.6%. These maximum reductions were achieved at optimal storage temperatures of 18.0–26.0 °C depending on the refrigerant. The results demonstrate that controlling the storage temperature based on domestic hot water demand share and space heating temperature is essential for optimal performance. The proposed system presents a promising pathway to significantly reduce electricity consumption in residential heating without relying on electric backup heaters.

## Abstract (web summary)

Conventional heat pumps often require electric heaters to meet domestic hot water demands because of their limited supply temperature. Zhibin Yu and colleagues report a quasi-two-stage flexible heat pump with in cycle thermal storage which provides space heating and domestic hot water through two operating modes, offering a practical solution for low-carbon residential heating.

## Introduction

The global imperative to achieve net-zero emissions has significantly accelerated the demand for energy-efficient technologies across all sectors, particularly in building heating systems. According to the International Energy Agency, a considerable share of worldwide energy consumption (50% in 2021) is converted to heat for buildings, industries, and other uses, with residential space heating (SH) and domestic hot water (DHW) alone comprising about 31% of total energy demand in Europe^1,2^. Despite the rising use of renewable energy sources for heating demand, fossil fuels remain the predominant energy source in many nations, contributing substantially to greenhouse gas emissions and undermining the worldwide aim of reaching net-zero emissions by 2050^3^. As a result, the electrification of heating systems, particularly through the widespread adoption of efficient heat pump technologies, is increasingly recognised as a key strategy for reducing fossil fuel dependence and achieving net-zero emissions targets consistent with the Paris Agreement.

Air-to-water heat pumps have gained recognition as an efficient and sustainable solution for residential heating, capable of providing both SH and DHW by utilising ambient thermal energy through integrated hot water systems that couple the hydronic heating system to circulate heated water via the building’s radiators or underfloor heating^4,5^. However, conventional single-stage systems often face limitations in delivering DHW at the high temperatures typically required ( ≥ 65 °C) to destroy legionella bacteria and mitigate microbiological risks^6,7^. This limitation typically necessitates the use of electric immersion heaters, which significantly increase energy consumption and reduce overall system efficiency^6,8^. Moreover, in modern well-insulated homes, the relative share of DHW demand is increasing, often exceeding that of SH, particularly during the warmer seasons^9,10^. Recent large-scale data from 45,000 UK households confirms that median DHW demand is approximately 90 l per day, and while DHW represents about 15% of total annual household heating energy, this proportion can rise to over 80% in the summer, highlighting the importance of dual-temperature heat supply systems^9^.

Previous studies have shown that DHW systems often operate with low efficiency due to thermal losses, suboptimal design, and hygiene-related temperature requirements. Boait et al. ^11^ demonstrated that instantaneous water heating can be more energy and carbon efficient than storage-based gas systems. Kropp et al. ^12^ highlighted the substantial energy losses in centralized DHW systems and emphasized the importance of reducing distribution temperatures and pipe lengths to improve heat pump performance. Graf et al. ^13^ further explored the trade-offs between efficiency, hygiene, and comfort, finding that decentralised or low-temperature DHW systems significantly reduce final energy demand but may pose hygiene risks if not properly managed. Meanwhile, Armstrong et al. ^7^ exposed the limitations of current storage tank designs in achieving sanitary conditions, noting that thermal stratification and off-peak heating can lead to bacterial growth and inefficient operation.

The core limitation in conventional heat pump systems arises from their diminished performance when operating across large temperature lifts. In single-stage heat pump cycles, delivering DHW at elevated temperatures requires substantial compression work, which not only increases energy consumption but also exacerbates irreversibilities due to higher throttling losses in the expansion process^14–16^. As the condenser temperature rises, the throttling loss rises and the coefficient of performance (COP) falls, meaning that more power is used, which makes high temperature heat pumps less efficient. To mitigate this, two-stage compression heat pumps have been developed, which divide the temperature lift between two sequential compression steps, thereby reducing the burden on each stage and enhancing cycle efficiency^17–19^. In a heat pump with two stages, the flash gas that is produced when the refrigerant expands is sent to a high-pressure compressor, keeping the flash gas from being compressed again in the low-pressure stage, which saves power and makes the COP higher. However, such systems often involve increased mechanical complexity, higher capital costs, and space requirements, making them less attractive for residential applications, particularly for retrofitting existing buildings where system footprint, noise, and installation constraints are critical considerations^20^.

Innovative solutions are therefore essential to improve high-temperature performance without adding excessive complexity^21^.

A recent thermodynamic analysis has demonstrated that flexible heat pump cycles incorporating thermal energy storage (TES) can achieve COP improvements thermodynamically equivalent to two-stage and enhanced vapour injection (EVI) architectures, up to 24% depending on refrigerant selection, while retaining the simplicity of a standard single-stage compressor^22^.

This performance enhancement is fundamentally linked to the recovery and utilisation of thermodynamic losses within the cycle. In particular, throttling in the expansion device represents a major source of irreversibility, where high-pressure refrigerant energy is dissipated during expansion. The flexible heat pump concept proposed by Yu et al. ^23^ addresses this limitation by integrating TES within the cycle, enabling the recovery of subcooling heat during charging and its reuse as a higher-temperature heat source during discharging.

Recent studies have explored the integration of TES with heat pumps to enhance flexibility and enable load shifting. For example, Du et al. ^24^ proposed a HP–PCM system that stores condensation heat during off-peak periods for later use in space heating, while Hu and Shen developed a multifunctional heat pump integrating TES within building envelopes to improve overall system performance^25^.

While these approaches mainly focus on load shifting and system multifunctionality, they do not directly address the thermodynamic challenge of high-temperature domestic hot water (DHW) production ( ≥ 65 °C).

Building upon prior research on flexible heat pump cycles with in-cycle TES, this study introduces a quasi-two-stage heat pump featuring dual parallel condensers to meet SH and DHW demands. Our work differs from existing integrated systems by introducing a configuration that decouples SH and DHW loops thermodynamically and temporally. Unlike the systems in the other sources, this design utilizes an integrated thermal storage unit to recover subcooling heat during the SH mode (charging) and employs it as a dynamic heat source for DHW production (discharging). This allows the system to efficiently reach the 65 °C temperatures required for DHW without relying on energy-intensive electric backup heaters. For this purpose, two parallel condensers are used in the system: when the heat pump is working in the charging mode, the first condenser used for space heating is activated at a lower temperature (40-50 °C). As soon as the heat storage has reached its maximum capacity, the heat pump switches to the discharging mode, where the first condenser is bypassed, and the second condenser operates at a higher temperature for DHW provision.

This study focuses on new-build residential dwellings, where DHW often accounts for a significantly larger proportion of total thermal demand compared to legacy housing, driven by improved building insulation and airtightness standards that reduce SH demand but leave DHW relatively constant^10^. This trend presents both a design challenge and an opportunity for optimizing dual-temperature energy systems that can efficiently meet both low- and high-temperature loads. A representative baseline scenario is evaluated using R290 as the refrigerant, operating with SH at 45 °C, DHW at 65 °C, and a total heating capacity of 6 kW, which aligns with realistic residential load profiles featuring a 15% DHW demand share. Furthermore, a comparative analysis involving seven refrigerants, R1234ze, R1234yf, R134a, R290, R407c, R600 (Isobutane) and R410a, is conducted across varying TES temperatures and DHW-to-SH demand ratios to assess trade-offs in COP improvement, energy savings potential, and environmental impact.

## Methodology

### Conventional heat pump

**Fig. 1:**
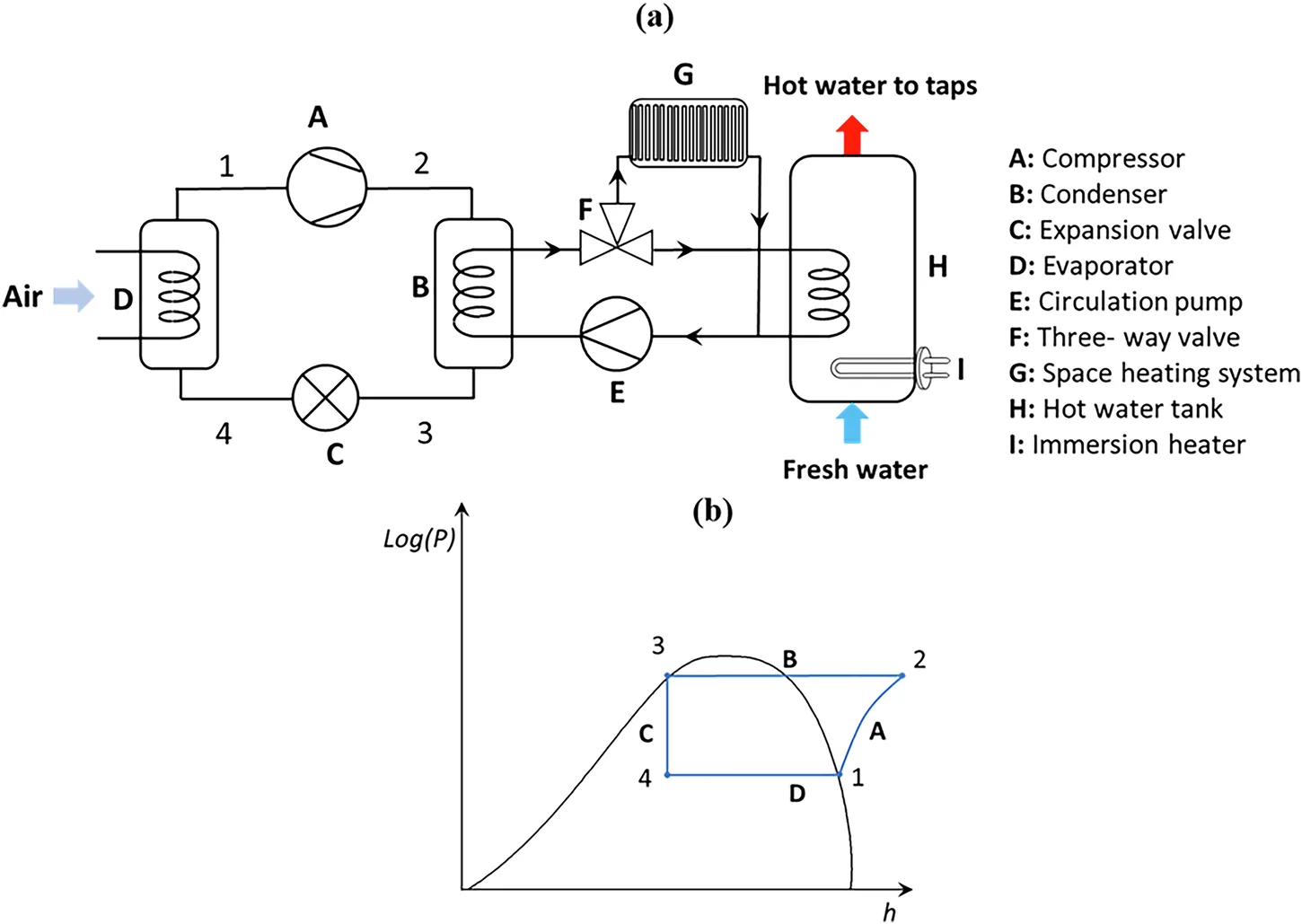
Conventional air source heat pump (ASHP) system and its operating principle. **a** Schematic diagram of a conventional ASHP system used for space heating (SH) and domestic hot water (DHW). **b** Pressure–enthalpy (P–h) diagram of the conventional ASHP cycle.

For comparison purposes, a standard air-source heat pump (ASHP) configuration commonly used in residential buildings is illustrated in Fig. 1a. In this system, the refrigerant absorbs heat from an ambient source—typically outdoor air—through the evaporator, where it vaporizes at low pressure. The resulting low-temperature vapour enters the compressor, where both its pressure and temperature are significantly increased. The high-pressure, high-temperature vapour then flows through the condenser, releasing heat to the water circuit during condensation. This thermal energy is subsequently used for SH and DHW supply. Following condensation, the refrigerant passes through an expansion valve, which reduces its pressure and temperature before it returns to the evaporator to begin the next cycle.

The ASHP system is typically split into an external unit, which extracts heat from outdoor air, and an internal unit, which includes backup heaters, control valves, temperature sensors, and circulation pumps. These components work together to regulate system operation and maintain required thermal comfort levels within the building. The heat delivery in a conventional system is divided into two primary pathways:

**SH:** Heated water, typically around 45 °C, is circulated to radiators or underfloor heating systems via a closed-loop central heating circuit. A feedback control system, linked to indoor temperature sensors, ensures stable SH performance.

**DHW:** Water for domestic use is heated indirectly through a heat exchanger connected to the thermal storage tank. The heat pump typically raises water temperature to approximately 45 °C. To comply with health regulations (e.g., Legionella control), an electric immersion heater is used to further increase the water temperature by around 20 °C, reaching the required 65 °C for safe hot water delivery. While this configuration enables moderate energy efficiency under standard conditions, its reliance on electric auxiliary heaters for DHW significantly reduces overall system performance, particularly during peak loads or colder ambient conditions. While the conventional system provides both SH and DHW, it relies on an electric immersion heater to meet DHW temperature requirements. This auxiliary device operates with a COP of approximately 1, significantly lowering the system’s overall efficiency. Moreover, if the heat pump is sized primarily for DHW provision, it can become oversized for SH, leading to off-design operation during most of the heating season and resulting in unnecessary capital cost and reduced part-load performance. To complement the schematic in Fig. 1a,  b presents the corresponding pressure–enthalpy (p–h) diagram, which illustrates the thermodynamic cycle of the refrigerant. This diagram helps visualize how energy is absorbed, compressed, released, and expanded throughout the system.

### Dual-mode flexible heat pump (DM-FHP)

As identified above in Section “Conventional heat pump”, there is a mismatch between the current heat pumps’ single supply temperature and the co-existing SH and SHW demands at different temperatures, which underpins the motivation for the dual-condenser flexible heat pump system described in Section “Dual-mode flexible heat pump (DM-FHP)”, which eliminates the need for immersion heaters and provides more efficient high-temperature DHW through TES-supported operation as described below.

**Fig. 2:**
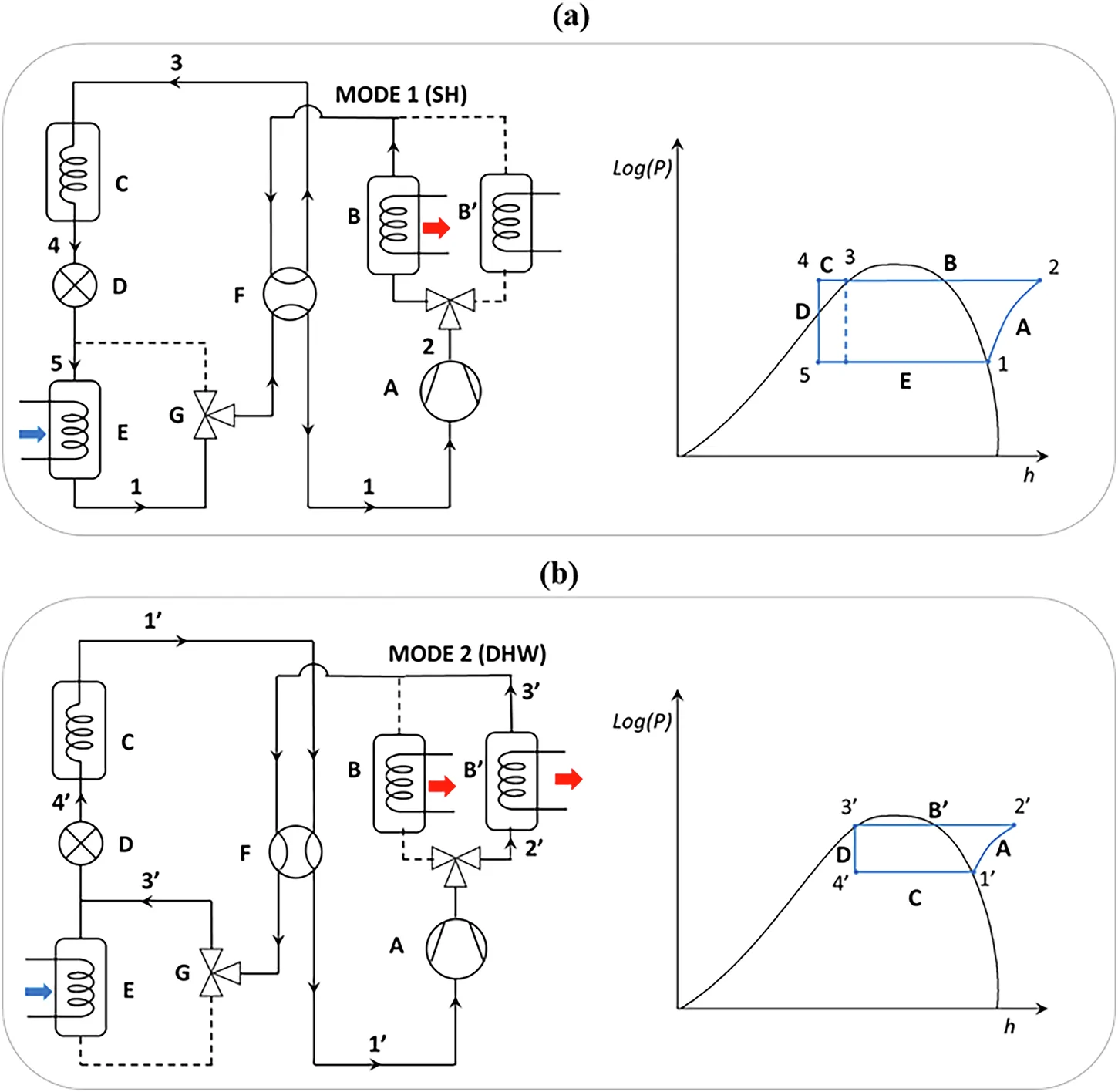
Schematic illustration of the dual-condenser flexible heat pump system with in-cycle TES. **a** Charging mode (Mode 1) with P-h diagram, where the heat pump delivers low-temperature heat for SH via Condenser B while simultaneously charging the TES; **b** discharging mode (Mode 2) with P-h diagram, in which stored thermal energy is utilized to supply high-temperature DHW via Condenser B′. A: Compressor; B: Condenser in SH mode; B’: Condenser in DHW mode; C: Thermal energy storage tank; D: Expansion valve; E: Evaporator; F: Four- way valve; G: Three-way valve.

The proposed dual-mode flexible heat pump system incorporates two parallel condensers to meet the both requirements of SH and DHW supply, as illustrated in Fig. 2. In this configuration, Condenser B is dedicated to SH, while the second condenser, B′, is positioned in parallel and assigned to DHW production. A three-way valve (G) enables switching between the two condensers based on the system’s operating mode.

During charging mode (Mode 1), the system prioritizes SH as shown in Fig. 2a. The refrigerant passes through Condenser B, delivering heat at a moderate temperature (e.g., at 45 °C) depending on the type and thermal characteristics of the property. Simultaneously, excess thermal energy carried by the hot liquid refrigerant exiting from the condenser is stored in an integrated TES unit. In this mode, Condenser B′ remains inactive. Once the TES is fully charged and hot water demand arises, the system switches to discharging mode (Mode 2) (see Fig. 2b). In this mode, the flow path is redirected: Condenser B is bypassed, and the refrigerant is routed through Condenser B′ to produce DHW at a higher output temperature, typically around 65 °C.

It should be noted that mode switching reliability is ensured through a ball valve arrangement with integrated check valves and a liquid receiver, as validated experimentally in the prototype implementation of this system^23^.

This configuration enables efficient delivery of high-temperature water by utilizing the stored thermal energy at a temperature much higher than the ambient air, thereby reducing the temperature lift required compared to conventional systems that must heat water directly from ambient conditions.

This dual-condenser configuration addresses key limitations in conventional heat pumps, namely, the reliance on electric booster heaters for DHW and performance degradation at high output temperatures. By decoupling SH and DHW supply modes and incorporating TES, the system could potentially achieve improved energy efficiency and greater operational flexibility.

#### Theoretical model for the dual condenser flexible heat pump

Propane (R290) is used as the refrigerant for the detailed comparison for a case study, and the effects of different refrigerants were subsequently analysed. For the selected case studied, the heat duty of the condenser was maintained at a constant value of 6 kW. Additionally, the evaporator temperature was fixed at 0 °C across all scenarios.

##### Space Heating Mode (charging mode)

Based on the thermodynamic properties determined at each point of the heat pump cycle, the mass flow rate during the space heating mode is calculated as follows:

1 $${\dot{m}}_{{SH}}=\frac{{\dot{Q}}_{{SH}}}{({h}_{2}-{h}_{3})}$$

For SH, the condenser (B′) outlet temperature is considered at 45 °C.

${\dot{Q}}_{{SH}}$ is the heat capacity of the condenser during the space heating mode. The condensing temperature is expressed as:

2 $${T}_{{cond},{SH}}={T}_{{water},{SH},{out}}+\Delta {T}_{{pinch}}{\prime},$$

$\Delta {T}_{{pinch}}{\prime}$ is the pinch temperature difference in the condenser. The condensers in both the SH and DHW loops are modeled using a fixed pinch temperature difference of 3.0 °C. This approach ensures that the refrigerant condensation temperature is always sufficiently higher than the required water delivery temperature (e.g., 48.0 °C for 45.0 °C SH) to facilitate effective heat transfer. Here evaporator is assumed to be an air to refrigerant heat exchanger with pinch temperature difference of $\Delta {T}_{{pinch}}$.

Evaporating temperature (refrigerant):

3 $${T}_{{eva},{SH}}={T}_{{air}.{out}}-\Delta {T}_{{pinch}}={T}_{{air}.{in}}-\Delta {T}_{{air}}-\Delta {T}_{{pinch}}$$

As a result, the evaporator effectiveness is defined as the ratio of the actual heat transferred from the air to the refrigerant to the maximum possible heat that could be transferred under ideal conditions.

4 $${\varepsilon }_{{eva}}=\frac{{Q}_{{actual}}}{{Q}_{\max }}$$

Where:

5 $${Q}_{{actual}}={\dot{m}}_{{air}}.{{cp}}_{{air},{in}}.({T}_{{air},{in}}-{T}_{{air},{out}})$$

and the maximum possible heat transferred to the refrigerant:

6 $${Q}_{\max }={\dot{m}}_{{air}}.{{cp}}_{{air},{in}}.({T}_{{air},{in}}-{T}_{{eva}})$$

As a result, we have:

7 $${\varepsilon }_{{eva}}=\frac{{T}_{{air}.{in}}-{T}_{{air}.{out}}}{{T}_{{air}.{in}}-{T}_{{eva},{SH}}}$$

Regarding the evaporator, a fixed effectiveness of 0.57 is assumed for the baseline case study. While heat exchanger effectiveness in practice varies with mass flow rate and air-side conditions, a fixed value is utilized here to isolate the thermodynamic benefits of the DM-FHP cycle architecture from component-level transient variations. This value (0.57) was selected based on established performance data for residential air-source heat pumps operating under standard rating conditions (e.g., 7 °C/3 °C air inlet/outlet). Furthermore, because this study focuses on a comparative analysis between the DM-FHP and a standard ASHP, both systems are modeled with the same evaporator characteristics. Any fluctuations in effectiveness due to climate variations would affect both systems similarly, meaning the relative COP improvements reported (e.g., 17.3%) remain a robust indicator of the cycle’s superior architecture. The influence of ambient air temperature on the maximum power reduction is further explored in Supplementary Fig. S1, which presents results at four ambient temperatures (0 °C, 5 °C, 7 °C, and 10 °C) under optimal TES temperature conditions.

The heat recovered by the TES can be determined using the mass flow rate in the SH mode, calculated from Eq. (1), as follows^23^:

8 $${{Q}}_{{charg},{SH}}={\dot{m}}_{{SH}}\left({h}_{3}-{h}_{4}\right)\Delta {t}_{{charge},{SH}}$$

In this scenario, the TES temperature (${T}_{{TES}}$) is between the evaporator and condenser temperatures. The compressor’s power consumption can be calculated as follows:

9 $${{W}}_{{comp},{SH}}={\dot{m}}_{{SH}}\left({h}_{2}-{h}_{1}\right)\Delta {t}_{{charge},{SH}}$$

where

10 $${h}_{2}={h}_{1}+\frac{\left({h}_{2{{{\rm{s}}}}}-{h}_{1}\right)}{{{{{\rm{\eta }}}}}_{{{{\rm{comp}}}}}}$$

where ${h}_{2s}$ is isentropic enthalpy in the outlet of compressor (from ${s}_{1}$ and ${P}_{2}$).

11 $${{W}}_{{comp},{SH}}=({\dot{m}}_{{SH}}\frac{\left({h}_{2{{{\rm{s}}}}}-{h}_{1}\right)}{{{{{\rm{\eta }}}}}_{{{{\rm{comp}}}}}})\Delta {t}_{{charge},{SH}}$$

The efficiency of the compressor ${{{{\rm{\eta }}}}}_{{{{\rm{comp}}}}}$ is modelled as a quadratic function of the pressure ratio ${R}_{p}$ and presented as follows, as demonstrated by Gullo et al. ^26^. and Dai et al. ^27^.

12 $${{{{\rm{\eta }}}}}_{{{{\rm{comp}}}}}=-0.0021{\left({{{{\rm{R}}}}}_{{{{\rm{p}}}}}\right)}^{2}-0.0155\left({{{{\rm{R}}}}}_{{{{\rm{p}}}}}\right)+0.7325$$

${R}_{p}$ is presented as:

13 $${R}_{p}=\frac{{P}_{2}}{{P}_{1}}$$

${P}_{2}$ and ${P}_{1}$ are the outlet and inlet pressure of the compressor, respectively in space heating mode. Irreversibilities within the condensers are accounted for by a 5% pressure drop to represent realistic flow resistance and its subsequent impact on compressor work:

14 $${P}_{3}={P}_{2}(1-0.05)$$

Finally, the COP during space heating mode can be calculated as:

15 $${{COP}}_{{FHP},{SH}}={{COP}}_{{standard},{SH}}=\frac{{{Q}}_{{SH}}}{{{W}}_{{comp},{SH}}}=\frac{{h}_{2}-{h}_{3}}{{h}_{2s}-{h}_{1}}$$

##### Domestic hot water mode

Once the TES is fully charged during space heating mode, the first condenser and primary evaporator are bypassed, and Condenser B′ and the TES are activated to produce hot water. The mass flow rate of refrigerant in the DHW mode is determined based on the thermodynamic properties at each point in the heat pump cycle, as follows:

16 $${\dot{m}}_{{DHW}}=\frac{{\dot{Q}}_{{DHW}}}{({h}_{2}{\prime} -{h}_{3}{\prime} )}$$

For DHW production, water temperature at the outlet of Condenser B′ is set at 65 °C. The heat rate discharged during the DHW supply is calculated as follows:

17 $${{Q}}_{{discharge},{DHW}}={\dot{m}}_{{DHW}}\left({h}_{1}^{{\prime} }-{h}_{5}^{{\prime} }\right)\triangle {t}_{{discharge},{DHW}}$$

The compressor’s power consumption during DHW operation can be calculated as follows:

18 $${{W}}_{{comp},{DHW}}={\dot{m}}_{{DHW}}\left({h}_{2}{\prime} -{h}_{1}{\prime} \right)\triangle {t}_{{discharge},{DHW}}$$

19 $${h{\prime} }_{2}={h{\prime} }_{1}+\frac{\left({h{\prime} }_{2{{{\rm{s}}}}}-{h{\prime} }_{1}\right)}{{{{{\rm{\eta }}}}{\prime} }_{{{{\rm{comp}}}}}}$$

20 $${{W}}_{{comp},{DHW}}=({\dot{m}}_{{DHW}}\frac{\left({h{\prime} }_{2{{{\rm{s}}}}}-{h{\prime} }_{1}\right)}{{{{{\rm{\eta }}}}{\prime} }_{{{{\rm{comp}}}}}})\Delta {t}_{{discharge},{DHW}}$$

21 $${\eta {\prime} }_{{comp}}=-0.0021{\left({R{\prime} }_{p}\right)}^{2}-0.0155\left({R{\prime} }_{p}\right)+0.7325$$

22 $${R{\prime} }_{p}=\frac{{P{\prime} }_{2}}{{P{\prime} }_{1}}$$

Same as charging mode we have:

23 $${P{\prime} }_{3}={P{\prime} }_{2}(1-0.05)$$

24 $${{COP}}_{{FHP},{DHW}}=\frac{{\dot{Q}}_{{DHW}}}{{\dot{W}}_{{comp},{DHW}}}=\frac{{\dot{m}}_{{DHW}}({h{\prime} }_{2}-{h{\prime} }_{3})}{{\dot{m}}_{{DHW}}\left({h{\prime} }_{2s}-{h{\prime} }_{1}\right)}=\frac{{h{\prime} }_{2}-{h}_{{\prime} 3}}{{h{\prime} }_{2s}-{h{\prime} }_{1}}$$

Operational time between discharging and charging modes

25 $$\frac{\triangle {t}_{{discharge},{DHW}}}{\triangle {t}_{{charge},{SH}}}=\frac{{\dot{Q}}_{{charg},{SH}}}{{\dot{Q}}_{{discharge},{DHW}}}$$

Given the time ratio, the total power consumption is expressed as follows:

26 $${W}_{{total}}={\dot{W}}_{{comp},{SH}.}\triangle {t}_{{charge},{SH}}+{\dot{W}}_{{comp},{DHW}}\triangle {t}_{{discharge},{DWH}}$$

The total heat provided is calculated as:

28 $${Q}_{{total}}={\dot{Q}}_{{SH}}\left(\triangle {t}_{{charge},{SH}}+\triangle {t}_{{discharge},{DHW}}\right)$$

Therefore, the average COP of the heat pump system can be calculated as

29 $${{COP}}_{{avg}}=\frac{{Q}_{{total}}}{{W}_{{total}}}$$

To calculate the COP improvement of the flexible heat pump as compared with the standard HP:

30 $${{COP}}_{{improvement}}=	\frac{{{{COP}}_{{avg}}-{COP}}_{{Standard}}}{{{COP}}_{{Standard}}}\cdot \left(100 \% \right)\\ =	\frac{{{{COP}}_{{standard},{SH}}-{COP}}_{{Standard}}}{{{COP}}_{{Standard}}}\cdot \left(100 \% \right)$$

The entire model was developed and run using MATLAB (Version: R2024a), integrated with the REFPROP v9.0 database. For more detailed information and the complete set of equations related to the flexible heat pump technology, please refer to our recent article by Yu et al. ^23^.

## Results and discussion

### A case study

To evaluate the performance of the proposed dual-condenser flexible heat pump system, a representative case study was selected based on realistic residential heating and hot water demand conditions in the UK climatic conditions. The system configuration assumes an air-source heat pump operating with propane as the refrigerant, selected for its favourable thermodynamic properties and low global warming potential (GWP). In this baseline scenario, the evaporator is maintained at 0  °C, and the air-side temperature difference is defined by a 7 °C inlet and 3 °C outlet. A 3  °C pinch point within the evaporator, with a heat exchanger effectiveness of 0.57. The spacing heating supply temperature is fixed at 45 °C. Domestic hot water is supplied at 65 °C, in compliance with Legionella prevention guidelines, without the use of an electric booster heater in the flexible system.

In the standard configuration, the heat pump delivers water at the same temperature used for space heating (45 °C), while the remaining temperature lift needed to reach DHW supply levels is provided by the booster heater. The share of DHW in the overall household heat load is taken as 15%, as a conservative assumption aligned with DESNZ (2024) analysis of 115,000 UK homes, which reports hot water at ~18% of domestic heat demand^9^. The total heating capacity in both systems is set as the same at 6 kW. The pressure drop across the heat exchangers is assumed to be 50 kPa. Heat transfer between the hot refrigerant and the storage medium is assumed to be imperfect, with a fixed temperature difference of 5 °C imposed to represent practical limitations in heat exchange. Table 1 summarizes the key operating parameters of both systems in the case study.

**Table 1:** Comparative analysis of a flexible heat pump and a standard heat pump supplemented by an immersion heater for domestic hot water provision in the selected case study

Parameter	Flexible heat pump	Standard heat pump
Refrigerant	Propane (R290)	Propane (R290)
Air Temperatures	7 °C inlet → 3 °C outlet	7 °C inlet → 3 °C outlet
Evaporator heat exchange effectiveness	0.57	0.57
Evaporator pinch point	3 °C	3 °C
Evaporating Temperature	0 °C	0 °C
Hot Water Output Temperature	65 °C	65 °C
Space Heating Temperature	45 °C	45 °C
DHW Load Share	15%	15%
Pressure Drop	50 kPa	50 kPa
Condensing Temperature (DHW Mode)	68 °C	n/a
Condensing Temperature (SH Mode)	48 °C	n/a
Condensing Temperature (Standard HP)	n/a	45 °C
Temperature boost by Immersion heater (Standard HP)	n/a	45 °C → 65 °C

Table 2 summarizes the performance metrics of the proposed quasi-two-stage flexible heat pump (FHP) with in-cycle TES, compared to a conventional heat pump system with booster electric heater. The analysis is based on a representative 60-minute charging mode for space heating followed by a discharging mode for domestic hot water, replicating typical daily SH and DHW demands. During the charging mode, the system stores thermal energy internally, recovering 4.32 kWh of heat into the TES at an average phase change material temperature of 27.5 °C. This recovered energy is later used during the discharging mode to provide DHW at elevated temperatures without requiring electric booster heating. The system operates against a baseline cold water inlet temperature of 15 °C, reflecting typical UK tap water conditions as reported in DHW consumption studies and device-based measurements by DESNZ^9,28^.

**Table 2:** Operational performance of a quasi-two-stage flexible heat pump with TES compared to a conventional system

	Flexible heat pump		Standard HP + Booster Heater	
	Operational Mode	Heat, Work, or COP	Operational Mode	Heat, Work, or COP
Space heating (SH)	Charging	32 (kWh)	Heat pump	32 (kWh)
Domestic hot water (DHW)	Discharging	5.6 (kWh)	Heat pump + Booster	5.6 (kWh)
Compressor work SH	Charging	8.53 (kWh)	Heat pump	8.53 (kWh)
Compressor work DHW	Discharging	1.42 (kWh)	Heat pump	0.90 (kWh)
Booster heater electricity	n/a	n/a	Booster heater	2.24 (kWh)
Heat extraction from air	Charging	27.79 (kWh)	Heat pump	23.47 (kWh)
	Discharging	4.18 (kWh)	Heat pump + booster	2.46 (kWh)
Heat recovery by the TES	Charging	4.32 (kWh)	n/a	n/a
Total Compressor Work	n/a	9.95 (kWh)	n/a	11.66 (kWh)
COP for SH	Charging	3.75	Heat pump	3.75
COP for DHW	Discharging	3.94	Heat pump + booster	1.78
${{COP}}_{{avg}}$ over 24 hours	n/a	3.78	n/a	3.22

In comparison with the standard system, which relies on a heat pump with electric immersion heater to raise the water temperature to meet DHW demands, the flexible heat pump achieves substantial energy savings. The total power consumption of the standard heat pump with electric immersion  heater across SH and DHW delivery is 11.66 kWh, while the proposed flexible heat pump requires only 9.95 kWh during both charging and discharging modes, leading to a power reduction of 14.7%. In terms of performance, the flexible heat pump delivers a high COP for DHW production at 3.94 due to its effective use of stored energy, compared to only 1.78 in the standard heat pump. Over a full 24-hour cycle, the flexible heat pump maintains an average COP of 3.78, which represents a 17.3% improvement over the standard heat pump’s COP of 3.22. Notably, both systems demonstrate the same COP of 3.75 during SH delivery, confirming that the performance advantage is attributed solely to the flexible heat pump’s superior DHW strategy.

Overall, the results demonstrate the energy-saving potential of the proposed quasi-two-stage operation of the flexible heat pump for both space heating and domestic hot water production at two distinct temperature levels.

### Impact of SH supply temperature on system performance

**Fig. 3:**
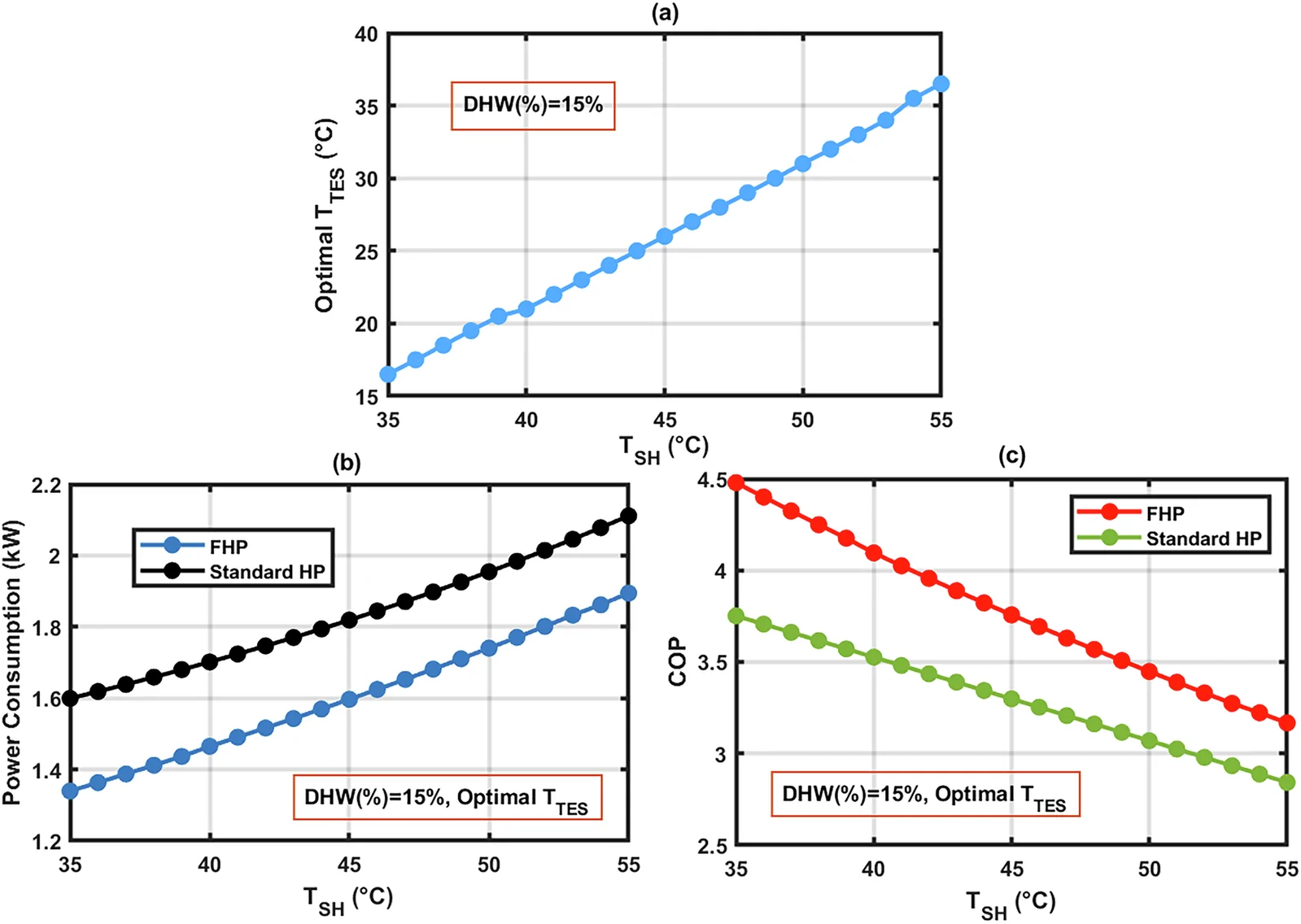
Performance comparison of the DM-FHP and the standard heat pump. **a** Optimal TES temperature at a 15% DHW share under varying SH supply temperatures. **b** Power consumption of the DM-FHP and the standard heat pump. **c** COP of the DM-FHP and the standard heat pump.

Figure 3 illustrates the performance of the DM-FHP system. It uses propane as the working refrigerant and assuming a 15% DHW share as the industry transits toward natural working fluids for heat pump and refrigeration. From this point onward, the results are presented assuming a thermal energy storage round-trip efficiency of 80%. In Fig. 3a, we see how the optimal ${T}_{{TES}}$ changes with variation of space heating supply temperatures (${T}_{{SH}}$). This trend exists because a lower TES temperature allows more heat to be recovered during the charging process (due to a higher temperature difference between the condenser and the TES), but it results in lower efficiency during discharging because the TES behaves like an evaporator at that lower temperature. On the other hand, a higher ${T}_{{TES}}$ improves performance during discharging (since the evaporator works at a higher temperature), but less heat is recovered during charging (we have more heat but in the lower temperature). Hence, for each ${T}_{{SH}}$ there is a balance point where this trade-off is optimized, that is why we see an optimal TES temperature at every ${T}_{{SH}}$ level.

Figure 3b, c compares the power consumption and COP of the DM-FHP and the standard heat pump with booster heater across different SH supply temperatures. As the ${T}_{{SH}}$ increases, the total power consumption of both systems rises, and their COPs decrease. However, we observe that the difference in performance between the two systems becomes smaller, in other words, their power and COP curves start to converge. This behaviour can be explained by the energy dynamics in both systems as ${T}_{{SH}}$ increases. In the DM-FHP, raising ${T}_{{SH}}$ results in greater heat recovery during the charging mode, allowing more energy to be stored in the TES. Consequently, more heat becomes available for use during discharging. However, since the DHW demand remains fixed, not all of the stored energy is actually needed or used. On the other side, in the standard heat pump, increasing ${T}_{{SH}}$ also raises the return water temperature from SH, which reduces the temperature lift required to reach the DHW supply level. As a result, the booster heater consumes less electricity. Therefore, at higher ${T}_{{SH}}$ values, the difference in performance between the DM-FHP and the standard heat pump becomes smaller, both in terms of COP and total power consumption.

**Fig. 4:**
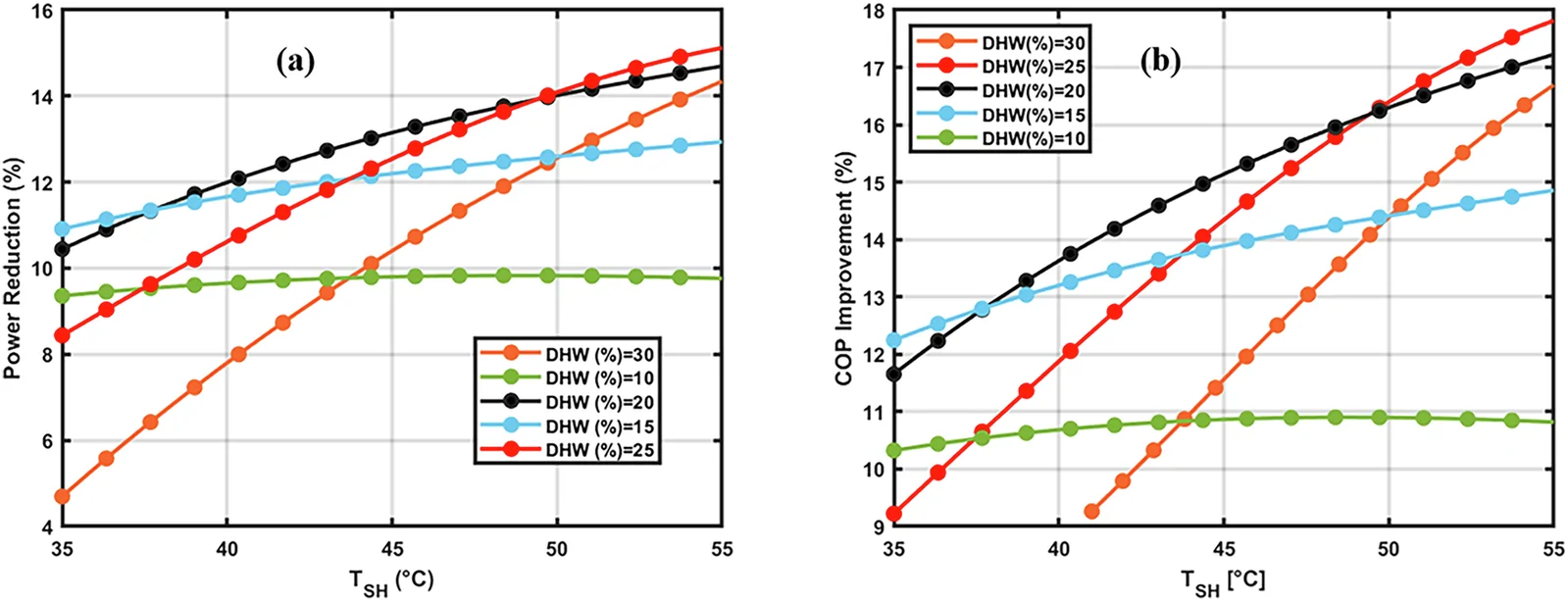
Impact of DHW share (%) on system performance across SH temperatures. **a** Power reduction (%) and **b** COP improvement (%) as a function of SH supply temperature, for different DHW shares.

Figure 4a, b shows the variation of power reduction and COP improvement of the DM-FHP system compared to the standard heat pump at different ${T}_{{SH}}$ values and when DHW share varies. As ${T}_{{SH}}$ increases, both power reduction and COP improvement generally increase; however, the slope of this improvement becomes smaller at higher ${T}_{{SH}}$ values. This is consistent with earlier findings according to Fig. 3b, c where the power consumption of the DM-FHP and the standard heat pump tend to converge at higher ${T}_{{SH}}$ values, as the additional heat stored in the TES is not fully utilized and the standard heat pump benefits from reduced booster heater usage, thereby reducing the relative improvement of the DM-FHP.

This trend is particularly noticeable at lower DHW shares such as 10%. In these cases, the DHW operation time is relatively short, and the amount of heat required for discharging can already be recovered at relatively low ${T}_{{SH}}$ values. As ${T}_{{SH}}$ increases, more heat is stored in the TES, but much of it remains unused because the DHW demand is already fully covered. In contrast, at higher DHW shares (e.g., 25% or 30%), more heat is required to support discharging. Therefore, a higher ${T}_{{SH}}$ is necessary to recover sufficient heat during charging, which shifts the point at which performance improvement (COP improve and power reduction) starts to slow down.

To help clarify this, consider the case where ${T}_{{SH}}$ is 35 °C and the DHW share is 10%, the required heat is relatively low (as compared to higher DHW share). In this case, the DM-FHP can recover and store more than enough heat at a high ${T}_{{SH}}$. When the DHW share increases to 15%, that same stored heat is still sufficient, so there is no need to adjust the TES temperature (or a bit lower ${T}_{{TES}}$). This leads to an increase in both COP improvement and power reduction because more of the stored heat is now being effectively used. However, as the DHW share increases further, say to 20% or more, the heat recovered at the previous ${T}_{{TES}}$ is no longer sufficient. To store more heat, the ${T}_{{TES}}$ must be reduced. While this increases the total stored energy, it comes at a lower temperature, which reduces its usefulness during discharging. As a result, performance gains start to decline at ${T}_{{TES}}$ of 35 °C. This illustrates why it is essential to analyse the interaction between ${T}_{{TES}}$, and DHW share. Each parameter affects the others, and a full understanding requires considering their combined influence, something explored further in the 2D contour results presented in the next section.

### Influence of ${T}_{{TES}}$ and DHW

**Fig. 5:**
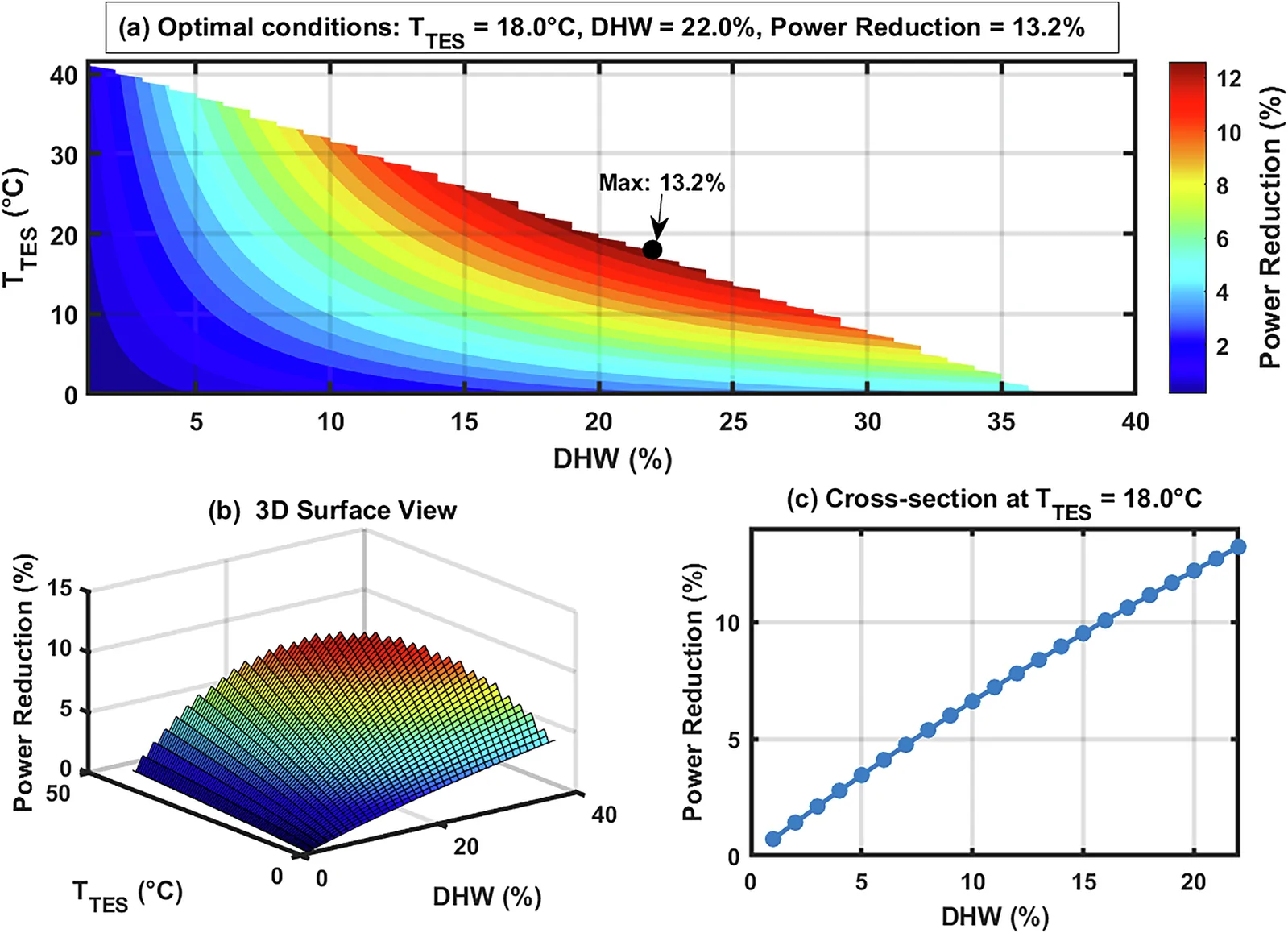
Combined effect of TES temperature and DHW demand share on power reduction of the DM-FHP system at a fixed space heating temperature of 45 °C. **a** 2D diagram showing power reduction as a function of TES temperature and DHW share, **b** combined impact of TES temperature and DHW share on power reduction shown from a 3D perspective, **c** power reduction in cross-section at optimal TES temperature when the DHW share is changing.

Figure 5 illustrates the power reduction performance of the DM-FHP at different TES temperatures and DHW shares, with ${T}_{{SH}}$ fixed at 45 °C. The 3D surface plot (bottom left) provides a broad view of how power reduction varies depending on both ${T}_{{TES}}$ and DHW share. This helps visualize the combined influence of these two parameters on system performance. To make the trend more readable, a 2D contour map (top) is also presented. In this view, the colour scale indicates the level of power reduction, red areas show higher reductions, while blue areas indicate lower performance. It can be observed that for each DHW share, a full range of power reduction values is available depending on the chosen ${T}_{{TES}}$ (with darker red indicating higher power reduction). Within each DHW level, there is a specific ${T}_{{TES}}$ that results in the highest power reduction, which is the optimal point for that operating condition. As shown in the figure, increasing the DHW share leads to a decrease in the optimal ${T}_{{TES}}$. When the DHW share is low, the required heat demand is also low, meaning the DM-FHP can store a smaller amount of heat, which allows for a higher ${T}_{{TES}}$—enabling discharging to operate at a higher effective heat source temperature. However, as the DHW share increases, more heat is required to meet the demand. In response, the DM-FHP must recover and store more energy during the charging cycle, which results in a lower optimal ${T}_{{TES}}$. As observed, the optimal DHW share and ${T}_{{TES}}$ are 22% and 18°C, respectively, resulting in the maximum power reduction of 13.2% when TSH is fixed at 45 °C.

The white areas in both the 3D surface and 2D contour plots represent conditions where the TES cannot recover enough heat to fully meet the DHW demand. This occurs when the ${T}_{{TES}}$ goes higher than the optimal ${T}_{{TES}}$ as it can be seen in the 2D contour plot, resulting in insufficient heat storage during charging. Similarly, when the DHW share exceeds around 36%, the heat required surpasses what the TES can deliver. In addition, the cross-sectional plot (bottom right) displays the power reduction at different DHW shares when the ${T}_{{TES}}$ is fixed at 18 °C (optimal ${T}_{{TES}}$). This helps highlight how performance improves as more of the stored heat is utilized—up to a certain point—before levelling off or declining depending on system limits.

Furthermore, the physical sizing of the TES is a critical design parameter that governs the system’s ability to balance intermittent space heating and DHW demands. The duration of the charging and discharging modes is fundamentally constrained by the energy balance between the heat recovered and the heat required for DHW, as expressed in the time-ratio relationship in Eq.( 25). In the representative case study, a 60-minute charging period results in 4.32 kWh of stored energy, which effectively supports a 15% DHW share (please see Table 2). However, as illustrated by the white areas in Fig. 5, there is a physical limit where the TES cannot recover enough heat to meet high DHW demands (above ~36% share). While increasing the TES size would allow the system to handle larger DHW loads, it would also increase the system’s footprint and mechanical complexity—factors that are often constraints in residential retrofitting. Moreover, if the TES is oversized for a specific SH/DHW ratio, the system can utilize active control sequences to repurpose surplus stored energy for space heating during the discharging phase, thereby ensuring the TES is fully utilized and further enhancing operational flexibility.

### Performance with different refrigerants

**Fig. 6:**
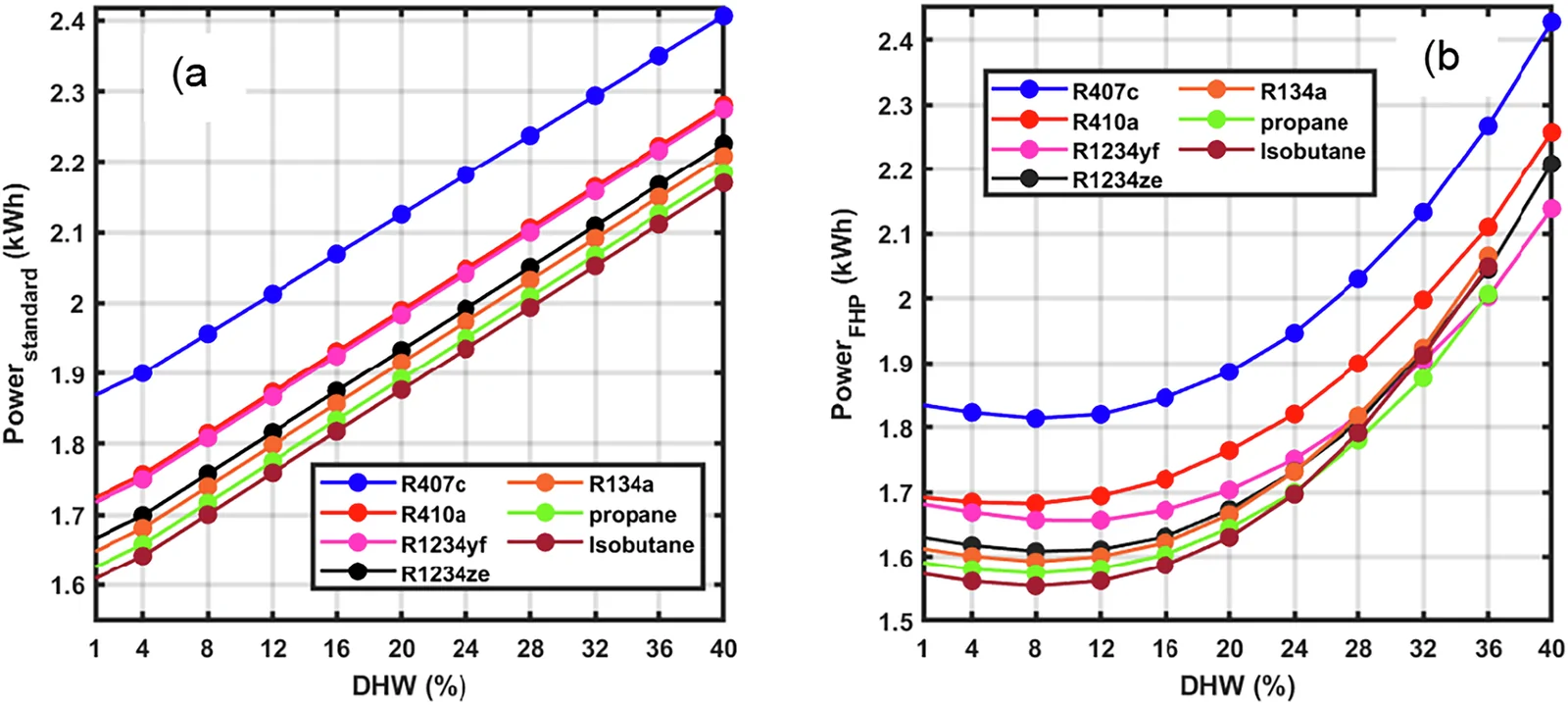
Power consumption comparison of the standard and flexible heat pumps. **a** Power consumption of the standard heat pump for various refrigerants across different DHW percentages. **b** Power consumption of the flexible heat pump for various refrigerants across different DHW percentages.

Figure 6a illustrates the power consumption of a standard heat pump as the DHW share increases (1–40%), at a given SH temperature of 45 °C, for various refrigerants. As the DHW share grows, a larger portion of the total heat demand is allocated to DHW production. Since the heat pump alone cannot supply water at the required 65°C DHW temperature, a greater contribution is needed from the booster heater. This leads to a consistent rise in power consumption across all refrigerants. Among them, refrigerants like R407c and R410a show the highest overall power use, while propane and isobutane are associated with lower power requirements.

Figure 6b illustrates the power consumption of the DM-FHP system as a function of DHW share for various refrigerants, with SH temperature fixed at 45 °C. As observed, the power consumption remains relatively low across all refrigerants when the DHW share is small, for example in the range of 4–8%. This is because, at lower DHW ratios, the total heat demand for DHW is low, so the DM-FHP can meet this demand using a smaller amount of recovered heat during the charging cycle. In these conditions, the TES can operate at a relatively high temperature, which serves as a higher-temperature heat source during discharging. This leads to reduced compressor work in the discharging mode and lower overall power consumption.

As the DHW share increases, however, the heat demand for DHW becomes more dominant relative to SH. To fulfil this higher demand using the TES, the DM-FHP must recover more heat during the charging phase. This increased recovery can only be achieved by reducing the ${T}_{{TES}}$, resulting in a lower effective temporary evaporator (or TES) temperature during discharging. Consequently, the system must work harder to supply DHW, causing a rise in compressor power consumption, especially beyond DHW shares of 20–25%. There is an intermediate range of DHW share, typically between 12% and 16%, where the DM-FHP achieves the most favourable balance between heat recovery, ${T}_{{TES}}$, and discharging effectiveness. In this region, the system operates efficiently across all refrigerants, avoiding both underutilization (as in very low DHW shares) and excessive compressor work (as in high DHW shares).

**Fig. 7:**
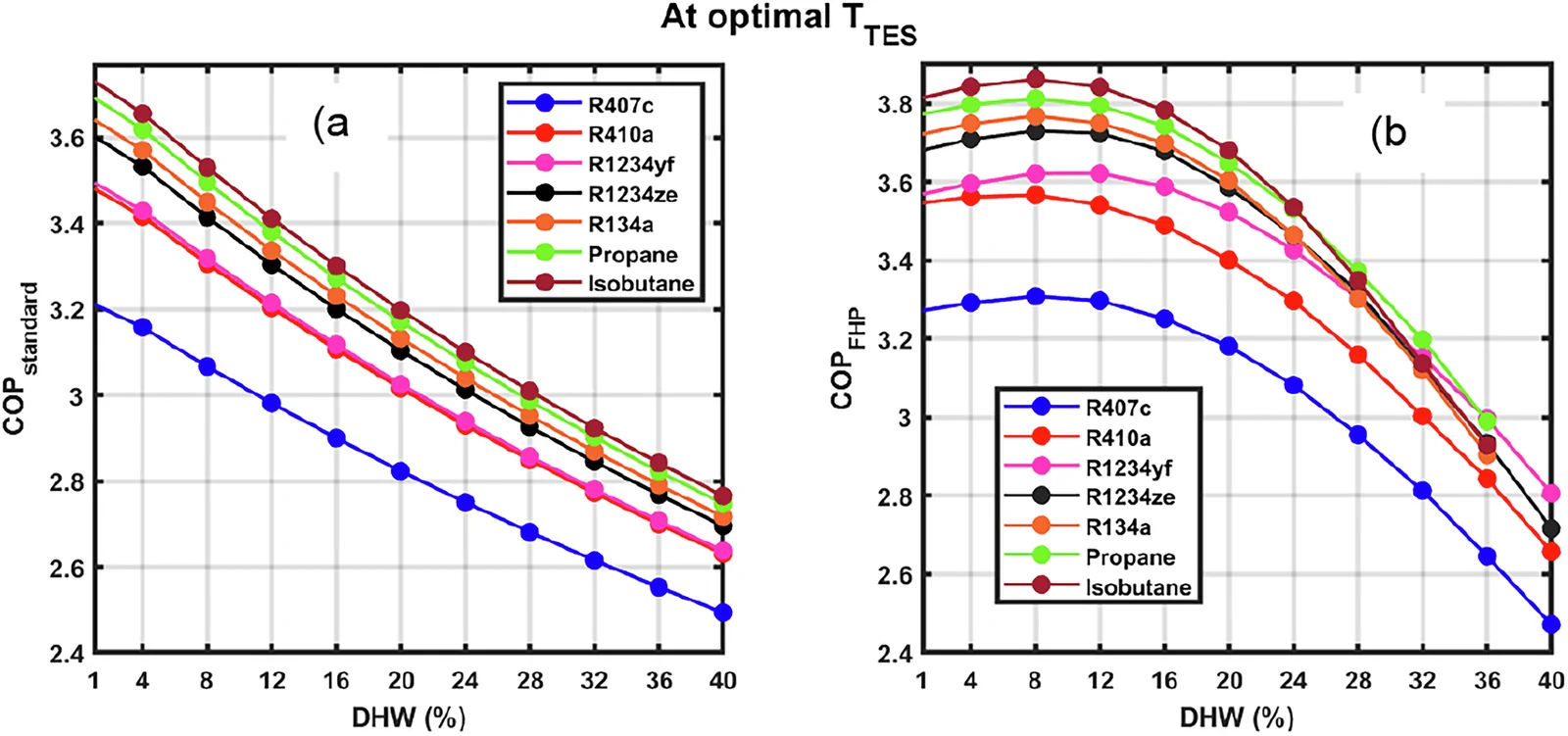
COP comparison of the standard and flexible heat pumps. **a** COP of the standard heat pump for various refrigerants across different DHW percentages. **b** COP of the flexible heat pump for various refrigerants across different DHW percentages.

Figure 7a, b presents the COP trends for the standard heat pump and the DM-FHP, respectively, across different DHW shares under the same conditions used in the power consumption analysis (${T}_{{SH}}=$45 °C) and different refrigerants at specific optimal ${T}_{{TES}}$. The underlying physical mechanism for these trends follows directly from the earlier discussion on power consumption: as electricity use increases, COP naturally decreases, and vice versa.

In the standard heat pump (Fig. 7a), COP decreases steadily with increasing DHW share for all refrigerants. This reflects the greater energy demand to meet higher DHW loads, particularly from the booster heater. In contrast, the DM-FHP (Fig. 7b) exhibits a peak COP at a moderate DHW share—typically between 12% and 16%, before declining. This corresponds to the minimum power consumption point previously identified, where the DM-FHP makes the most efficient use of TES charging and discharging.

Among all refrigerants, isobutane and propane demonstrate the highest COPs in both systems, while R407c consistently shows the lowest, reinforcing their relative performance advantages under different operating conditions.

**Fig. 8:**
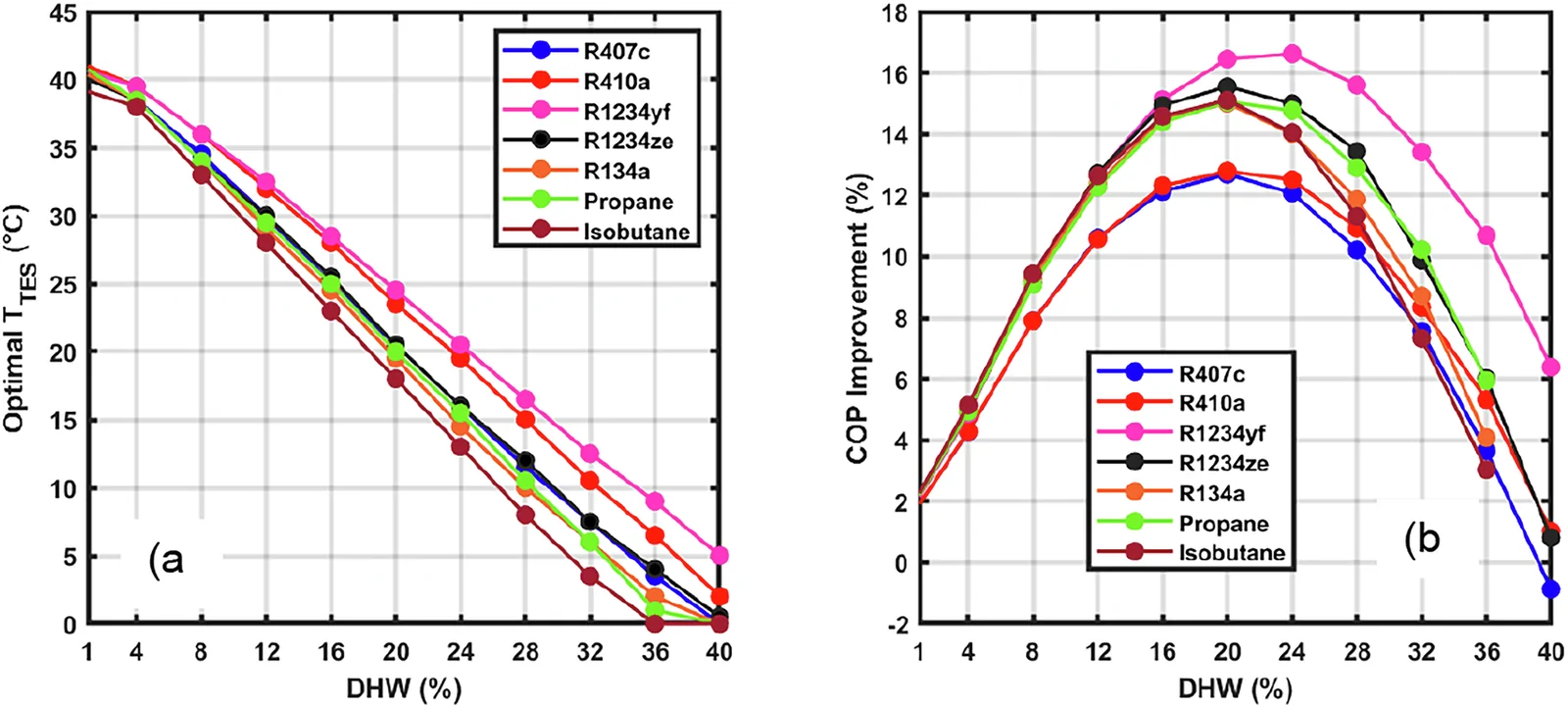
Performance characteristics of the flexible heat pump. **a** Optimal TES temperature versus DHW percentage for different refrigerants. **b** COP improvement versus DHW percentage for different refrigerants.

Figure 8a shows how the optimal ${T}_{{TES}}$ varies with DHW share for different refrigerants, while Fig. 8b presents the corresponding COP improvement of the DM-FHP compared to the standard HP. For each DHW ratio, the COP improvement is calculated based on its associated optimal ${T}_{{TES}}$ from Fig. 8a. The COP improvement curves in Fig. 8b exhibit a clear peak for all refrigerants. At lower DHW shares, the required heat is minimal, allowing discharging to occur at higher TES temperatures. While this enables efficient operation, the overall contribution of discharging to system performance is small. At higher DHW shares, more heat is required, but the TES must operate at lower temperatures to meet this demand, reducing discharging efficiency. Importantly, this dome-shaped trend in COP improvement can also be explained by the interplay between DM-FHP and standard HP power consumption: the standard HP’s COP decreases steadily with increasing DHW share, while the DM-FHP shows a maximum at a specific DHW level. As a result, the performance gap between the two systems is maximized at an intermediate DHW share, creating a clear peak in COP improvement.

The optimal DHW share that maximizes COP improvement differs slightly by refrigerant. For example, R1234yf reaches its peak improvement at 24%, indicating its suitability for applications with higher DHW demand, while other refrigerants like propane and R410a show their best performance around 22% and 20% DHW share, respectively. This analysis highlights that choosing the appropriate refrigerant and maintaining a suitable DHW share are the important factors in optimizing DM-FHP performance. Although the TES temperature is not shown in this figure, its influence has been demonstrated in earlier results and should also be considered alongside DHW share and refrigerant type.

While R1234yf and R1234ze demonstrate the highest power reduction potential, their relatively low volumetric heating capacity (VHC) must be considered for practical residential applications. Refrigerants with lower VHC require larger compressor displacement and piping to deliver the same heating capacity, which can increase system size and cost. Considering this trade-off, propane was selected as the representative refrigerant for detailed analysis in the preceding sections, as it provides a balanced combination of energy efficiency and volumetric capacity while also aligning with low-GWP requirements.

**Fig. 9:**
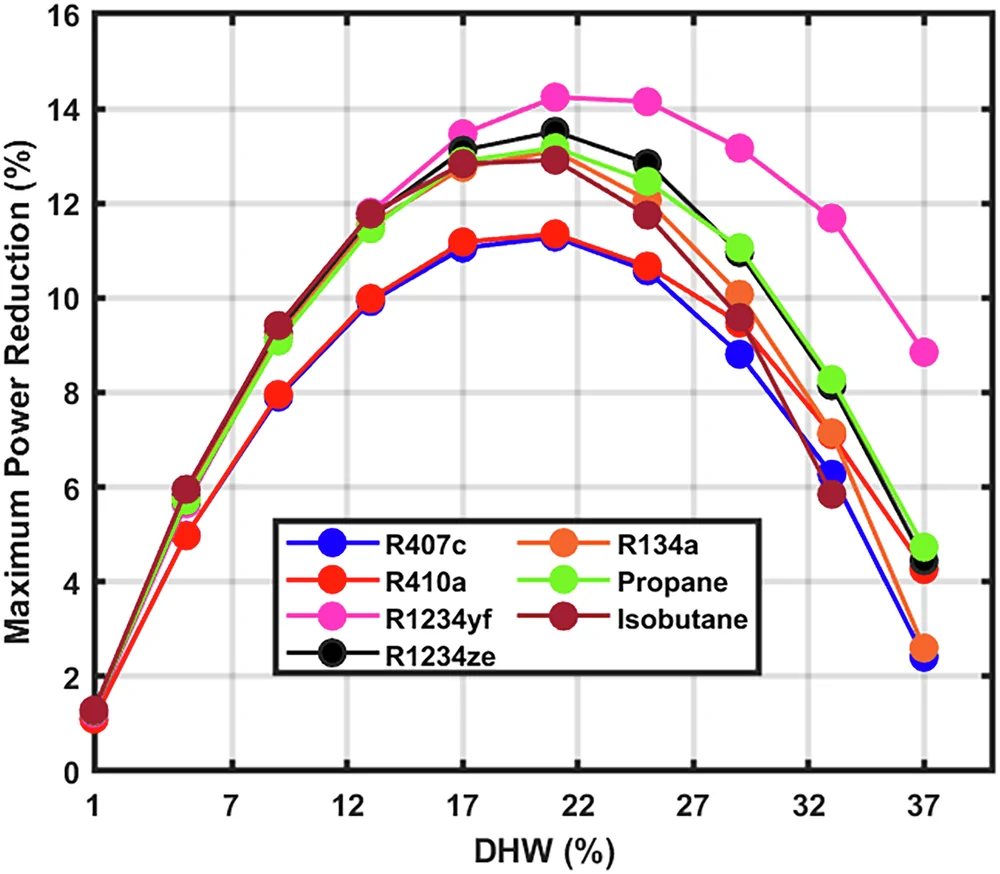
Maximum power reduction achieved by the DM-FHP relative to a standard heat pump across varying DHW shares for seven refrigerants. The results compare seven refrigerants (R407c, R410a, R1234yf, R1234ze, R134a, Propane, and Isobutane) across DHW shares.

Figure 9 shows the maximum power reduction (%) achieved by the DM-FHP relative to a standard heat pump across different DHW shares for a range of refrigerants. Each curve displays a clear peak, indicating the optimal DHW share at which the highest power savings occur. For most refrigerants, this optimal point falls around a DHW share of 21%, reflecting a balanced interaction between charging and discharging in the DM-FHP system. At this stage, the DM-FHP performs most efficiently, with R1234yf achieving the highest power reduction of about 14.2%, followed by R1234ze at approximately 13.5%. Propane and R134a both deliver close to 13.2%, while isobutane reaches around 12.9%. R410a and R407c follow with reductions of about 11.4%.

Among all refrigerants, R1234yf stands out not only for reaching the highest peak in power reduction but also for sustaining relatively high performance over a wider range of DHW shares. Even beyond the optimal point, R1234yf maintains a stronger reduction level compared to others, especially in higher DHW ratios. This indicates that R1234yf may be better suited for scenarios where DHW demand is consistently high, offering more energy savings. In contrast, R407c and R410a show their peak COP improvement at lower DHW shares compared to the other refrigerants. This indicates that these refrigerants perform best when the domestic hot water demand is relatively low in DM-FHP.

**Fig. 10:**
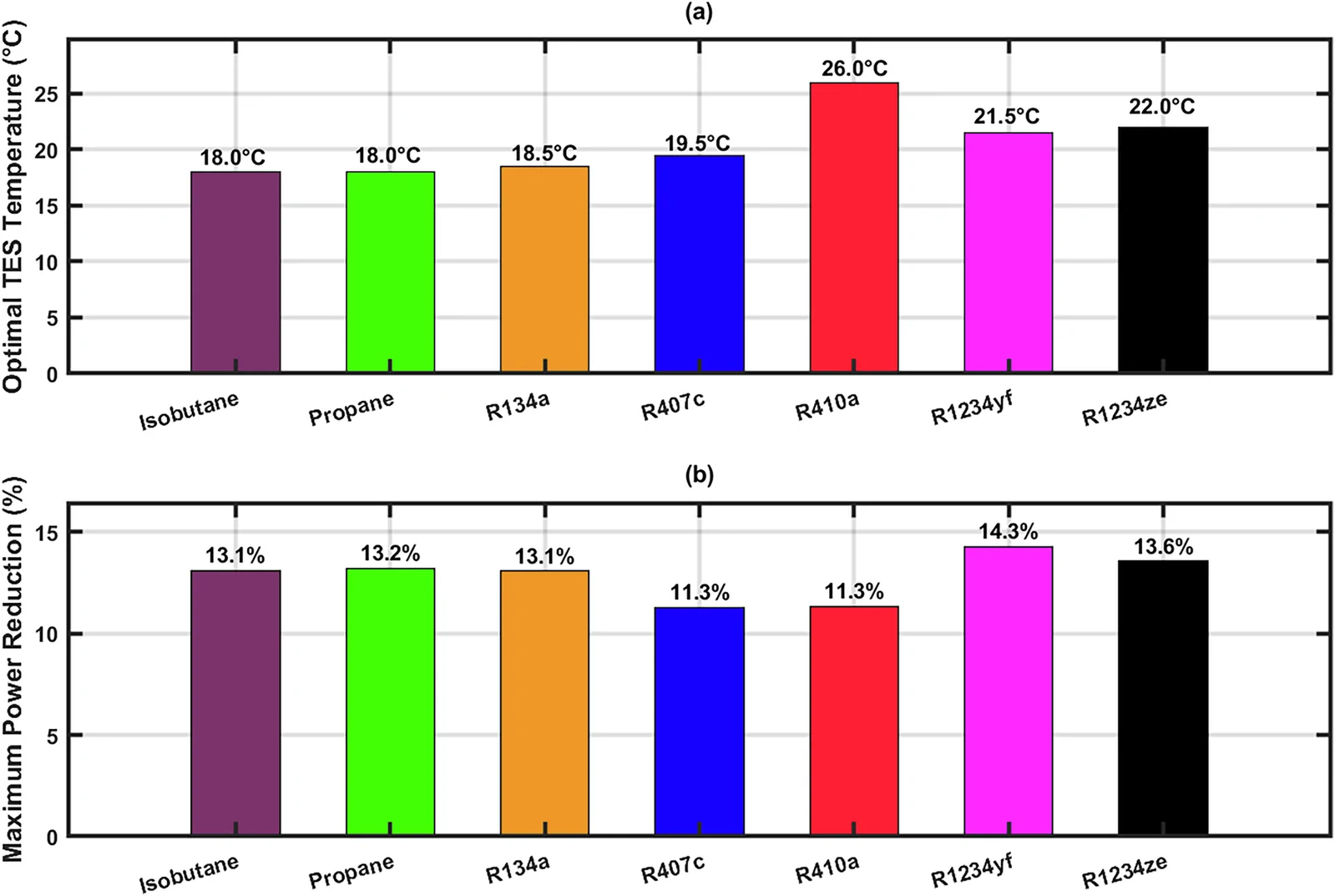
Optimal TES temperature and corresponding maximum power reduction for seven refrigerants at a fixed space heating temperature of 45 °C. **a** The optimal ${T}_{{TES}}$ corresponding to each refrigerant, **b** the associated maximum power reduction at that optimal ${T}_{{TES}}$ for each refrigerant.

Figure 10 provides a concise summary of the maximum power reduction achieved by the DM-FHP system for each refrigerant at a fixed SH temperature of 45 °C. The upper graph displays the optimal ${T}_{{TES}}$ required to achieve the maximum power reduction for each refrigerant at a fixed SH temperature of 45 °C. This clearly highlights how each refrigerant performs best at a different TES setpoint, for instance, R410a operates most efficiently at 26.0 °C, while isobutane and propane achieve their best results at a lower ${T}_{{TES}}$ of 18.0 °C.

The lower graph complements this by showing the corresponding maximum power reduction (%) that can be achieved across all DHW shares. Among the refrigerants, R1234yf delivers the highest reduction at 14.3%, followed closely by R1234ze (13.6%), propane (13.2%), and R134a (13.1%). In comparison, R407c and R410a offer the lowest reductions, both around 11.3%. These results emphasize the importance of maintaining a refrigerant-specific ${T}_{{TES}}$ to unlock the full energy-saving potential of the DM-FHP system. This combined view clearly highlights the need to carefully select and control the ${T}_{{TES}}$ depending on the refrigerant used. It also reinforces how refrigerant choice and TES settings work together to maximize the energy savings potential of the DM-FHP system.

Although the results in Fig.10 suggest that R410a and R1234yf and R1234ze are betters choses from the thermodynamic performance perspective, propane is used for our case studies and parametric analysis above because the heat pump industry are in the process to transit from synthetic refrigerant to natural working fluids such as hydrocarbons.

### Impact of TES heat transfer and efficiency on DM-FHP performance

In reality, the effectiveness of heat transfer from the refrigerant coil to the TES medium is not 100%, meaning there is always a temperature difference between the refrigerant outlet and the TES. In previous analyses, this temperature difference (ΔT) was assumed to be 5 °C.

**Fig. 11:**
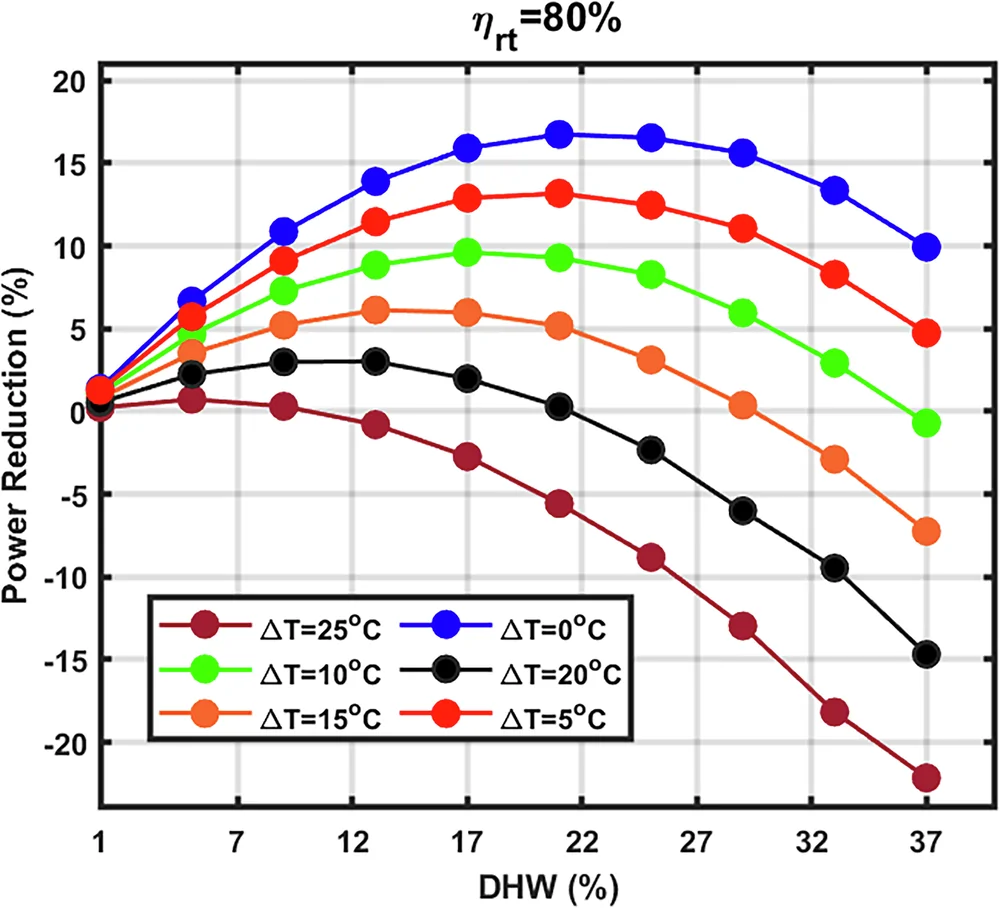
Effect of heat transfer effectiveness on DM-FHP power savings. The figure explores how different ΔT values affect the power reduction percentage at different DHW loads.

However, in practice, many PCM materials exhibit hysteresis, where melting and solidification occur over different temperature ranges. This behaviour effectively increases the temperature lift by requiring a higher charging temperature and a lower discharging temperature, which can reduce system efficiency. The sensitivity analysis presented in Fig. 11, where ΔT is varied from 0 to 25 °C, provides an indirect assessment of this effect. As shown in the Fig. 11, this analysis explores how different ΔT values affect the power reduction percentage of the DM-FHP compared to a standard HP across a range of DHW shares. In this diagram, we assume ${{{{\rm{\eta }}}}}_{{rt}}$ of the TES is 80% when the ${T}_{{SH}}$ is set to 45 °C for propane as the refrigerant.

Additionally, ΔT between the refrigerant and the TES is considered the same during both charging and discharging modes. Moreover, for each DHW share and ΔT, there is a specific optimal TES temperature that must be identified to achieve the highest power reduction percentage. As ΔT increases, representing reduced heat transfer effectiveness, the power reduction percentage of the DM-FHP steadily declines. For instance, in the ideal case where ΔT = 0 °C, the system achieves a maximum power reduction of approximately 16.9%. This gradually drops as ΔT increases, falling to around 13.2% at ΔT = 5 °C, matching the baseline case used in earlier analyses, and ultimately reaching only 0.7% at ΔT = 25 °C.

These results emphasize that selecting PCM materials with minimal hysteresis and ensuring high heat transfer effectiveness are critical for maintaining the 13.2% power savings identified in our baseline case. In addition to the reduced savings, the optimal DHW share (i.e., the share at which power reduction peaks) also shifts significantly with ΔT. As heat transfer becomes less effective, the system reaches its maximum benefit at progressively lower DHW shares. Specifically, the optimal DHW share drops from 23% at ΔT = 0°C to just 5% at ΔT = 25°C. For each ΔT value (0, 5, 10, 15, 20, and 25°C), the corresponding optimal DHW shares are 23%, 22%, 18%, 14%, 11%, and 5%, respectively. This shift highlights how weaker thermal interaction between the refrigerant coil and TES not only limits the value of energy savings, but also limits the operating conditions under which the DM-FHP outperforms a standard HP. These results highlight that lower thermal resistance between the refrigerant coil and TES improves heat transfer, allowing the flexible heat pump to perform more efficiently under varying DHW demands.

**Fig. 12:**
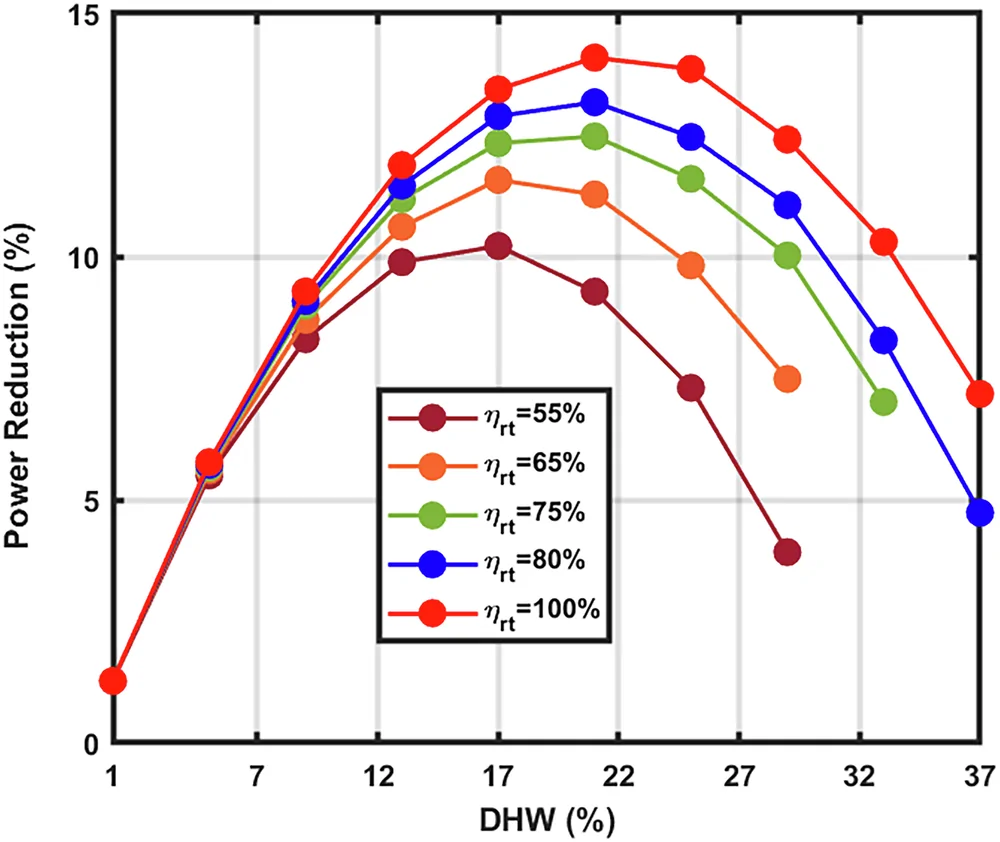
Effect of round-trip efficiency on power reduction of the DM-FHP system across varying DHW demand shares. The round-trip efficiency ranges from 55 to 100%, with higher values consistently leading to greater power savings.

Figure 12 evaluates how the round-trip efficiency ${{{{\rm{\eta }}}}}_{{rt}}$ of the TES affects overall power reduction across varying DHW shares. While previous simulations assumed ${{{{\rm{\eta }}}}}_{{rt}}$ = 80%, this analysis compares system performance across a broader range: 55% to 100%. As expected, higher round-trip efficiency consistently yields greater power savings. At ${{{{\rm{\eta }}}}}_{{rt}}$ = 100%, the DM-FHP system achieves a maximum power reduction of nearly 14.5% at around 22% DHW share, reflecting idealised storage conditions with no thermal degradation. As ${{{{\rm{\eta }}}}}_{{rt}}$ decreases, both the magnitude of peak savings and the range of DHW shares over which savings are realised decline significantly. For example, at ${{{{\rm{\eta }}}}}_{{rt}}$ = 80%, power reduction peaks around 13.2%, while at ${{{{\rm{\eta }}}}}_{{rt}}$ = 55%, it falls to just above 10% and drops sharply after 22% DHW share. This highlights the importance of achieving high TES efficiency in practical system designs. Losses in storage—whether due to poor insulation, inadequate exchanger effectiveness, or material degradation—can significantly erode the benefits of the flexible heat pump concept. To maximise system performance, TES components should aim for ${{{{\rm{\eta }}}}}_{{rt}}$ levels above 75%, particularly in applications with high or variable DHW demands

It should be noted that the present study is based on a steady-state thermodynamic model with a rule-based operation sequence and fixed heating capacity. In practical applications, heat pumps must operate under highly transient space heating loads and stochastic domestic hot water demand profiles. While the current analysis demonstrates the thermodynamic viability and power reduction potential of the proposed system, the temporal decoupling of SH and DHW modes introduces inherent intermittency. Maintaining indoor thermal comfort and stable DHW supply temperatures under dynamic conditions would require advanced control strategies. Therefore, future work should focus on coupled system simulations integrating transient building and DHW demand profiles, enabling optimisation of TES sizing and refinement of control strategies for real-world operation.

During summer, when space-heating demand is negligible or absent, both operating modes of the flexible heat pump, charging and discharging, can be employed for domestic hot water (DHW) production.

Because summer ambient temperatures are substantially higher than those encountered during winter operation, the temperature lift required for DHW production is significantly reduced in charging mode, where heat is upgraded directly from the ambient air to the DHW supply temperature of 60–65 °C. Consequently, the charging mode operates more efficiently than in winter, a benefit that is also observed in conventional heat pumps.

However, the flexible heat pump offers an additional advantage. Thermal energy recovered and stored during charging mode can subsequently be utilised through discharging mode to provide DHW at 60–65 °C. As in winter operation, the discharging mode benefits from a lower effective temperature lift and can therefore achieve a higher COP than a conventional heat pump producing DHW directly from the ambient air, even under summer conditions.

Consequently, the flexible heat pump is expected to maintain its performance advantage during summer operation. While both systems benefit from elevated ambient temperatures, the flexible heat pump can further exploit stored thermal energy through its high-efficiency discharging mode, resulting in improved overall efficiency and superior performance for DHW production.

## Conclusion

This study presented a performance evaluation of a dual-mode flexible heat pump system equipped with integrated thermal energy storage to meet both space heating and domestic hot water demands without relying on electric booster heaters. The system using popular refrigerant propane was analysed under realistic operating conditions, with a supply water temperature of 45 °C for space heating and 65 °C for domestic hot water. In the baseline case using propane, a thermal energy storage round-trip efficiency of 80% and a 5 °C temperature approach between the refrigerant and thermal storage were assumed. Under these conditions, the system achieved a power reduction of 13.2% compared to a conventional heat pump.

Comprehensive parametric analyses were conducted to explore the influence of domestic hot water share, space heating temperature, and thermal storage temperature. Results revealed that the optimal thermal storage temperature decreases with increasing domestic hot water demand, while higher space heating temperatures shift the optimal point upward. The best-performing refrigerants—R1234yf, R1234ze, propane, and isobutane—achieved power reductions between 13.0% and 14.3% when optimally configured.

Further analysis demonstrated that performance can be further enhanced by improving heat transfer effectiveness and round-trip efficiency of the thermal energy storage. For example, reducing the temperature approach between the refrigerant and storage from 5 to 0 °C raised the power savings from 13.2 to 16.9%, highlighting the importance of strong thermal integration. Similarly, higher round-trip efficiency enables more of the recovered heat to be effectively reused, especially under varying domestic hot water loads.

Looking forward, the system has untapped potential: when domestic hot water demand is low, the remaining stored heat could be repurposed for space heating during discharging, enabling even greater performance and flexibility. This highlights the importance of advanced control strategies and dynamic operation. Therefore, future work will focus on coupled system simulations with realistic load profiles and control strategies to further validate system performance under practical conditions.

## Supplementary information

Transparent Peer Review file

Supplementary Information

## Data Availability

The datasets generated and/or analysed during the current study are available within the paper. Other materials and data are available from the corresponding author, Z.Y., upon reasonable request.

## Code availability

The code developed for this study is available from the corresponding author upon reasonable request, subject to consideration of intellectual property and ongoing research and commercialisation activities.
