# Supplementary material for: A quasi-two-stage heat pump for efficient dual—temperature supply—space heating and domestic hot water
Source: Commun Eng. 2026 Jul 21;5:194. doi: 10.1038/s44172-026-00739-9 (PMC13754852; doi:10.1038/s44172-026-00739-9)
Supplement: Supplementary file 1 [file 44172_2026_739_MOESM1_ESM.pdf]

nature portfolio

Peer Review File

A quasi-two-stage heat pump for efficient dual-temperature supply – space heating and domestic hot water

Corresponding Author: Professor Zhibin Yu

This file contains all reviewer reports in order by version, followed by all author rebuttals in order by version.

Version 0:

Reviewer comments:

Reviewer #1

(Remarks to the Author)
This manuscript introduces a quasi-two-stage heat pump for both space heating and domestic hot water. By integrating a latent thermal energy storage unit in the refrigerant cycle, the heat pump can recover heat from subcooling and charge the thermal storage material during space heating at lower supply temperature, and discharge the stored heat for effective domestic hot water heating at higher supply temperature. The system's effectiveness is evaluated with simulation on several refrigerant candidates and domestic hot water and space heating load and temperature scenarios.

The authors did a good job describing the configuration and operation of the proposed system and offered simulated case study and parametric analysis on a few design and operation parameters. Meanwhile, the lack of discussion on some critical design and operation parameters such as the sizing of the TES and its charging/discharging control leaves significant gaps in the analysis. Furthermore, with the intrinsic intermittent operation between space heating and DHW, whether and how the system can simultaneously meet both varying heating load in a realistic setup remains under-discussed. Therefore, a major revision is recommended to allow the authors revise and add necessary discussion for publication in this Journal.

My detailed comments are below:

a. The authors might want to include the following publications that also describe multi-temperature heat pumping with thermal energy storage in the literature review and compare with the proposed system:

Hu, Y., & Shen, B. (2025). Development and evaluation of a multi-functional heat pump with embedded thermal storage. Applied Thermal Engineering, 267, 125862.

Du, R., Wu, M., Wang, S., Wu, S., Wang, R., & Li, T. (2024). Integrated heat pump with phase change materials for space heating. Renewable and Sustainable Energy Reviews, 203, 114769.

b. The compressor icon (A) in Figure 1 (a) should be reverted so that the heat exchanger connected with the circulation loop (B) is a condenser in the refrigerant cycle. It might also be helpful if the state points in the P-h diagram can be marked on the system schematic at the corresponding locations, similar to the way in Figure 2.

c. A fixed melting point is assumed for the PCM, with a 5 degC fixed temperature difference assumed for heat transfer. In reality, many PCM materials demonstrate hysteresis where melting starts above the theoretical melting point and freezing starts below it. The degree of hysteresis obviously varies depending on materials and conditions, but its effect could lead to higher charging temperature and lower discharging temperature, which will hurt the system efficiency and thus warrants some discussion.

d. The proposed system provides space heating and DHW intermittently, following a rule-based operation sequence that fully charges TES in space heating mode, then switches to provide DHW until TES is depleted. Therefore, the size of the TES is a critical design parameter: a sufficiently sized TES enables more energy storage during SH period, and thus allows better performance at higher DHW load. The sizing of the TES needs to be discussed on top of the existing discussion on the super heat temperature. Moreover, if the TES is oversized for certain SH/DHW ratio, an active control of charging and discharging can still fulfill both SH and DHW need.

e. In reality the heat pump needs to meet the space heating and DHW load simultaneously, and thus simply assuming a constant 6kW heating output along with the abovementioned rule-based operation sequence might not necessarily guarantee that the space temperature is maintained under varying building load, as well as the DHW supply temperature is met under typical water drawing schedules. It’s good that the author explored different DHW load ratio trying to discuss different operating scenarios. However, due to the intrinsic intermittency in the system operation, system simulation coupled with transient and realistic space and water heating loads is necessary to demonstrate the proposed system's viability and operation characteristic.

Reviewer #2

(Remarks to the Author)
The study proposes a system that utilizes thermal energy storage to simultaneously supply space heating water and domestic hot water. It aims to improve system efficiency by reducing the pressure ratio required when producing high-temperature domestic hot water. The paper compares the effects of various factors—including refrigerant type and intermediate temperature—on overall performance. The following points should be considered for further revision:
1.Although staged heating via two steps can enhance compressor efficiency and thus improve heat pump performance, the two heat exchange processes with the thermal storage medium will increase the suction-discharge pressure difference between the two heating stages. This could lead to an actual degradation in system performance. The paper insufficiently accounts for heat exchanger temperature differences in its comparisons, and experimental validation of real-world performance is needed.
2.In practice, systems with vapor-injection that also carry space heating loads offer greater potential. A comparative analysis should be included.
3.Refrigerant liquid through the four-way valve will significantly reduce switching reliability. This issue should be addressed in the system design.
4.How will the system operate during summer?
5.The analysis indicates that while HFO refrigerants offer higher energy efficiency, their low volumetric cooling capacity limits their practical applicability in this type of residential hot water system.
6.The manuscript text requires further proofreading. For example: The three-way valve is labeled G, not H.

Reviewer #3

(Remarks to the Author)
In their paper "A quasi-two-stage heat pump for efficient dual-temperature supply - space heating and domestic hot water" submitted to Communications Engineering, the authors propose a new dual-mode heat pump system that integrates latent thermal energy storage inside the unit to handle both space heating and domestic hot water more efficiently. In one mode it heats the space while charging the storage, and in the other it uses the stored heat to produce higher-temperature domestic hot water, reducing the need for electric backup heaters.

The authors develop a comprehensive quasi steady first law thermodynamic model to quantify the energy and power savings achievable with the proposed dual mode configuration. The model relies on: (i) a compressor efficiency that depends solely on the instantaneous pressure ratio; (ii) a fixed effectiveness for the evaporator unit; and (iii) a fixed pinch point temperature difference in the condenser (to the best of my understanding).

Using this framework, the paper evaluates system performance for various refrigerants and operating conditions. The results indicate that the proposed system can reduce electricity consumption by approximately 13 to 14% with a conventional baseline system, with R1234yf yielding the highest power reduction among the refrigerants considered. A key insight is that optimal control of the thermal energy storage temperature, as a function of both the DHW demand share and the space heating temperature, is crucial to maximizing the efficiency gains of the dual-mode operation.

Overall, the manuscript is well structured, the methodology is clearly laid out, and the results are convincingly presented and discussed. The topic is timely and relevant for residential energy systems, and the proposed concept has clear practical significance. I therefore recommend acceptance after minor revisions. My specific suggestions to further strengthen the manuscript are listed below.

1) Clarification and justification of modelling assumptions. The modelling assumptions and simplifications would benefit from a more detailed and transparent discussion. In particular, it is not entirely clear how the condenser is modelled in terms of exergy destruction (or heat exchanger effectiveness). Please clarify whether a specific heat exchanger model (e.g., epsilon-NTU, LMTD with a fixed pinch, or a simplified approach) is used to represent the condenser and how irreversibilities are accounted for. The assumption of a fixed evaporator effectiveness over the full range of operating conditions needs to be better justified. Since evaporator performance can vary with mass flow rate, air-side conditions, and refrigerant state, please discuss the implications of this simplification on the accuracy and robustness of the results (particularly important if several climate conditions are to be explores). If available, consider adding a brief sensitivity analysis (even qualitative) or referencing experimental/analytical studies that support the chosen effectiveness value.

2) Influence of climate and ambient conditions. The current analysis appears to focus on a limited set of ambient air conditions. To better assess the value of the proposed concept, it would be beneficial to explore different climates. I encourage the authors to investigate the potential savings across a range of outdoor air temperatures (and, if feasible,

humidities), representative of different climate zones. Even a parametric study using typical winter design temperatures for a few reference climates (e.g., mild, moderate, cold) would significantly strengthen the conclusions and highlight where the dual mode system provides the greatest benefit.

3) Economic perspective: CAPEX vs OPEX. While the thermodynamic and electrical performance benefits are clearly demonstrated, the paper does not discuss the additional investment cost associated with implementing the dual mode heat pump and integrated latent thermal energy storage. The authors are strongly encouraged to provide at least indicative capital cost estimates for the proposed system relative to a standard single mode heat pump, even if these estimates come with wide uncertainty bands. A simple comparison of incremental CAPEX to expected OPEX savings (based on the reported electricity reductions and a reasonable electricity price) would greatly enhance the practical relevance of the work and help readers gauge the payback potential. If detailed cost data are unavailable, a qualitative discussion supported by literature-based cost ranges for thermal storage and additional components would still be very useful.

With these minor revisions and clarifications, the manuscript will provide an even stronger and more comprehensive contribution to the literature on efficient residential heat pump systems for combined space heating and domestic hot water production.

Version 1:

Reviewer comments:

Reviewer #1

(Remarks to the Author)
The authors have fully addressed my comments.

Reviewer #3

(Remarks to the Author)
I am very pleased with the thorough response of the authors and the significant efforts that they put into this revised manuscript. The modelling assumptions are now clearly stated and enough information is provided to replicate the findings or build upon this innovative and promising solution. I thus recommend to accept this manuscript for publication, as I believe that it will be very valuable to the research on air- as well as ground-source heat pumping systems.

Open Access This Peer Review File is licensed under a Creative Commons Attribution 4.0 International License, which permits use, sharing, adaptation, distribution and reproduction in any medium or format, as long as you give appropriate credit to the original author(s) and the source, provide a link to the Creative Commons license, and indicate if changes were made.
In cases where reviewers are anonymous, credit should be given to 'Anonymous Referee' and the source.
The images or other third party material in this Peer Review File are included in the article’s Creative Commons license, unless indicated otherwise in a credit line to the material. If material is not included in the article’s Creative Commons

license and your intended use is not permitted by statutory regulation or exceeds the permitted use, you will need to obtain permission directly from the copyright holder.
To view a copy of this license, visit https://creativecommons.org/licenses/by/4.0/

Response to Reviewers
Manuscript Title: A quasi-two-stage heat pump for efficient dual-temperature supply – space heating and domestic hot water
Manuscript ID: COMMSENG-25-0770
Authors: Zahra Hajabdollahi Ouderji, Muhammad Abid, Zhibin Yu

Dear Editor and Reviewers,

We are grateful to all three reviewers for their thorough and constructive evaluation of our manuscript. Their feedback has significantly strengthened the work. In preparing the revised manuscript and this response letter, the following key changes have been made:

- **Expanded Technical Discussion:** Detailed sections on TES sizing, control logic, and system operational limits have been added to the revised manuscript, including a discussion of the physical boundaries governing the intermittent SH/DHW operation. The steady-state scope of the present study is explicitly acknowledged, with dynamic simulation under realistic transient loads identified as future work. The abstract has also been condensed to approximately 200 words.
- **Climate and Ambient Analysis:** A parametric study on the influence of ambient air temperature (0°C to 10°C) has been conducted and presented in the response letter (Figure R1), demonstrating that the system provides the greatest efficiency benefit in colder climates where conventional heat pumps are most penalised.
- **Modelling Transparency:** All key modelling assumptions have been clarified in the revised manuscript, including fixed pinch point temperature differences for condensers, evaporator effectiveness justification, pressure drop treatment, and compressor efficiency correlation.
- **Practical Reliability and Implementation:** Concerns regarding mode switching reliability have been addressed by reference to the experimentally validated prototype [Yu et al., 2023], which employs a ball valve arrangement to eliminate liquid refrigerant passage through switching valves. A clarifying note has been added to the revised manuscript accordingly.
- **Comparative Context:** The proposed system has been positioned relative to enhanced vapour-injection (EVI) cycles in the revised manuscript to highlight its thermodynamic performance equivalence and mechanical simplicity. Regarding economic analysis, this falls outside the defined scope of the present thermodynamic study, as acknowledged in the revised manuscript, and is identified as an important direction for future work.

Below, we provide a point-by-point response to each of the reviewers' comments, with specific references to the changes made in the revised manuscript. We believe these revisions significantly strengthen the manuscript and hope it is now suitable for publication in *Communications Engineering*.
Sincerely,

Point-by-point reply to the Reviewers' comments:

Reviewer #1 (Remarks to the Author):

This manuscript introduces a quasi-two-stage heat pump for both space heating and domestic hot water. By integrating a latent thermal energy storage unit in the refrigerant cycle, the heat pump can recover heat from subcooling and charge the thermal storage material during space heating at lower supply temperature, and discharge the stored heat for effective domestic hot water heating at higher supply temperature. The system's effectiveness is evaluated with simulation on several refrigerant candidates and domestic hot water and space heating load and temperature scenarios.

The authors did a good job describing the configuration and operation of the proposed system and offered simulated case study and parametric analysis on a few design and operation parameters. Meanwhile, the lack of discussion on some critical design and operation parameters such as the sizing of the TES and its charging/discharging control leaves significant gaps in the analysis. Furthermore, with the intrinsic intermittent operation between space heating and DHW, whether and how the system can simultaneously meet both varying heating load in a realistic setup remains under-discussed. Therefore, a major revision is recommended to allow the authors revise and add necessary discussion for publication in this Journal.

My detailed comments are below:

a. The authors might want to include the following publications that also describe multi-temperature heat pumping with thermal energy storage in the literature review and compare with the proposed system:

Hu, Y., & Shen, B. (2025). Development and evaluation of a multi-functional heat pump with embedded thermal storage. Applied Thermal Engineering, 267, 125862.

Du, R., Wu, M., Wang, S., Wu, S., Wang, R., & Li, T. (2024). Integrated heat pump with phase change materials for space heating. Renewable and Sustainable Energy Reviews, 203, 114769.

Reply: We thank the reviewer for suggesting these highly relevant publications. We have now included both Hu and Shen (2025) and Du et al. (2024) in our revised Introduction as follows:

Recent studies have explored the integration of TES with heat pumps to enhance flexibility and enable load shifting. For example, Du et al. [24] proposed a HP–PCM system that stores condensation heat during off-peak periods for later use in space heating, while Hu and Shen developed a multifunctional heat pump integrating TES within building envelopes to improve overall system performance [25].

While these approaches mainly focus on load shifting and system multifunctionality, they do not directly address the thermodynamic challenge of high-temperature domestic hot water (DHW) production (≥65 °C).

Building upon prior research on flexible heat pump cycles with in-cycle TES, this study introduces a quasi-two-stage heat pump featuring dual parallel condensers to meet SH and DHW demands. Our work differs from existing integrated systems by introducing a configuration that decouples SH and DHW loops thermodynamically and temporally. Unlike the systems in the other sources, this design utilizes an integrated thermal storage unit to recover subcooling heat during the SH mode (charging)

and employs it as a dynamic heat source for DHW production (discharging). This allows the system to efficiently reach the 65°C temperatures required for DHW without relying on energy-intensive electric backup heaters.

b. The compressor icon (A) in Figure 1 (a) should be reverted so that the heat exchanger connected with the circulation loop (B) is a condenser in the refrigerant cycle. It might also be helpful if the state points in the P-h diagram can be marked on the system schematic at the corresponding locations, similar to the way in Figure 2.

Reply: Thank you for the helpful comment. The compressor icon in Figure. 1 has been corrected to ensure that component (B) properly represents a condenser in the cycle. In addition, the state points from the P–h diagram have been added to the system schematic at the corresponding locations to improve clarity.

c. A fixed melting point is assumed for the PCM, with a 5 degC fixed temperature difference assumed for heat transfer. In reality, many PCM materials demonstrate hysteresis where melting starts above the theoretical melting point and freezing starts below it. The degree of hysteresis obviously varies depending on materials and conditions, but its effect could lead to higher charging temperature and lower discharging temperature, which will hurt the system efficiency and thus warrants some discussion.

Reply: Thank you for this insightful comment. We agree that PCM hysteresis and supercooling can affect system performance by increasing the effective temperature lift during charging and discharging. In the revised manuscript, we have clarified this aspect in Section 3.5 by explicitly linking it to our existing sensitivity analysis of the temperature difference (ΔT), which was already included in the original manuscript. This analysis, where ΔT is varied from 0 °C to 25 °C, effectively captures the thermodynamic impact of hysteresis, showing a reduction in power savings from 16.9% to 0.7% as the effective temperature gap increases. We have also added a brief discussion highlighting the importance of selecting PCM materials with low hysteresis to maintain system performance as follows:

However, in practice, many PCM materials exhibit hysteresis, where melting and solidification occur over different temperature ranges. This behaviour effectively increases the temperature lift by requiring a higher charging temperature and a lower discharging temperature, which can reduce system efficiency. The sensitivity analysis presented in Fig. 11, where ΔT is varied from 0 °C to 25 °C, provides an indirect assessment of this effect.

And as follows:

These results emphasize that selecting PCM materials with minimal hysteresis and ensuring high heat transfer effectiveness are critical for maintaining the 13.2% power savings identified in our baseline case.

d. The proposed system provides space heating and DHW intermittently, following a rule-based operation sequence that fully charges TES in space heating mode, then switches to provide DHW until TES is depleted. Therefore, the size of the TES is a critical design parameter: a sufficiently sized TES enables more energy storage during SH period, and thus allows better performance at higher DHW load. The sizing of the TES needs to be discussed on top of the existing discussion on the

super heat temperature. Moreover, if the TES is oversized for certain SH/DHW ratio, an active control of charging and discharging can still fulfill both SH and DHW need.

Reply: Thank you for this insightful comment regarding the importance of TES sizing. We agree that TES capacity is a key design parameter governing the system's ability to meet higher DHW loads. In the revised manuscript, we have added a discussion in Section 3.3 to clarify that TES capacity defines the temporal decoupling limits of the system, as reflected by the "white regions" in Figure 5 where the storage becomes insufficient to meet DHW demand. We also linked the charging/discharging time ratio to the stored energy (e.g., 4.32 kWh in the baseline case) and discussed how increasing TES size can extend system capability, while noting practical constraints such as system footprint.

In addition, as suggested, we added a note that when surplus heat is available, the system can be controlled to repurpose that energy for SH during the discharging mode, preventing energy underutilization and increasing total system efficiency.

The following text has been added to the revised manuscript:

Furthermore, the physical sizing of the TES is a critical design parameter that governs the system's ability to balance intermittent space heating and DHW demands. The duration of the charging and discharging modes is fundamentally constrained by the energy balance between the heat recovered and the heat required for DHW, as expressed in the time-ratio relationship in Eq. 25. In the representative case study, a 60-minute charging period results in 4.32 kWh of stored energy, which effectively supports a 15% DHW share (please see Table 2). However, as illustrated by the white areas in Fig. 5, there is a physical limit where the TES cannot recover enough heat to meet high DHW demands (above ~36% share). While increasing the TES size would allow the system to handle larger DHW loads, it would also increase the system's footprint and mechanical complexity—factors that are often constraints in residential retrofitting. Moreover, if the TES is oversized for a specific SH/DHW ratio, the system can utilize active control sequences to repurpose surplus stored energy for space heating during the discharging phase, thereby ensuring the TES is fully utilized and further enhancing operational flexibility.

e. In reality the heat pump needs to meet the space heating and DHW load simultaneously, and thus simply assuming a constant 6kW heating output along with the abovementioned rule-based operation sequence might not necessarily guarantee that the space temperature is maintained under varying building load, as well as the DHW supply temperature is met under typical water drawing schedules. It's good that the author explored different DHW load ratio trying to discuss different operating scenarios. However, due to the intrinsic intermittency in the system operation, system simulation coupled with transient and realistic space and water heating loads is necessary to demonstrate the proposed system's viability and operation characteristic.

Reply: Thank you for this valuable comment. We would like to clarify that the present study already considers a range of operating scenarios by varying the DHW load share (1%–40%), allowing us to assess system performance under different demand conditions and identify its operational limits (e.g., the "white regions" in Fig. 5 where TES capacity becomes insufficient).

The intermittency of the system is addressed through the temporal decoupling framework (Eq. 25), which balances the heat recovered during space heating with the heat required for DHW.

We agree, however, that full validation under realistic and transient load conditions requires dynamic system simulation. This limitation has now been explicitly acknowledged at the end of the Discussion section, and a brief statement has been added to the Conclusion outlining this as future work. The following text has been added to the revised manuscript:

It should be noted that the present study is based on a steady-state thermodynamic model with a rule-based operation sequence and fixed heating capacity. In practical applications, heat pumps must operate under highly transient space heating loads and stochastic domestic hot water demand profiles. While the current analysis demonstrates the thermodynamic viability and power reduction potential of the proposed system, the temporal decoupling of SH and DHW modes introduces inherent intermittency. Maintaining indoor thermal comfort and stable DHW supply temperatures under dynamic conditions would require advanced control strategies. Therefore, future work should focus on coupled system simulations integrating transient building and DHW demand profiles, enabling optimisation of TES sizing and refinement of control strategies for real-world operation.

And:

This highlights the importance of advanced control strategies and dynamic operation. Therefore, future work will focus on coupled system simulations with realistic load profiles and control strategies to further validate system performance under practical conditions.

Reviewer #2 (Remarks to the Author):

The study proposes a system that utilizes thermal energy storage to simultaneously supply space heating water and domestic hot water. It aims to improve system efficiency by reducing the pressure ratio required when producing high-temperature domestic hot water. The paper compares the effects of various factors—including refrigerant type and intermediate temperature—on overall performance. The following points should be considered for further revision:

1. Although staged heating via two steps can enhance compressor efficiency and thus improve heat pump performance, the two heat exchange processes with the thermal storage medium will increase the suction-discharge pressure difference between the two heating stages. This could lead to an actual degradation in system performance. The paper insufficiently accounts for heat exchanger temperature differences in its comparisons, and experimental validation of real-world performance is needed.

Reply: Thank you for this important comment. We would like to clarify that the proposed system is not a conventional two-stage heat pump. The Dual-Mode Flexible Heat Pump (DM-FHP) employs a single compressor, and the term "quasi-two-stage" refers to the temporal and thermodynamic decoupling of space heating (SH) and domestic hot water (DHW) modes, rather than sequential compression stages. Therefore, the system does not introduce additional compression stages that would increase the overall pressure lift. On the contrary, during the discharging mode, the TES acts as a higher-temperature heat source (typically 18–26 °C), which increases the effective evaporation temperature and reduces the compressor pressure ratio compared to a conventional air-source heat pump operating at low ambient conditions.

We agree that the impact of heat exchanger temperature differences is important and can influence system performance. In the manuscript, this effect is considered through the sensitivity analysis of ΔT (Section 3.4, Fig. 11), where the influence of increasing temperature difference on system performance is discussed.

We also agree that experimental validation is essential. The underlying flexible heat pump concept used in this work has been previously proposed and experimentally validated in a single-condenser configuration (Yu et al., Ref [23]). The present study extends this experimentally validated concept to a dual-condenser architecture tailored for combined SH and DHW applications.

2. In practice, systems with vapor-injection that also carry space heating loads offer greater potential. A comparative analysis should be included.

Reply: Thank you very much for your insightful comment. We agree that vapor-injection (VI) systems represent an important benchmark for improving heat pump performance under high temperature lift conditions.

We would like to clarify that such configurations have already been systematically compared in our previous work (Yu et al., 2023), where a unified thermodynamic framework was used to evaluate flexible (quasi-two-stage) heat pump cycles alongside advanced layouts, including two-stage and injection-based systems. These configurations incorporate subcooling and flash gas removal mechanisms, which are the fundamental principles behind enhanced vapor-injection (EVI) cycles.

That study demonstrated that the flexible heat pump concept is thermodynamically comparable to these injection-based systems under equivalent conditions. Therefore, the present work does not repeat a full comparative analysis, but instead builds upon that established thermodynamic equivalence to develop and analyse a specific system architecture (DM-FHP) for combined space heating and domestic hot water applications.

To make this connection clearer, we have added a reference to this previous study in the Introduction, along with a brief discussion positioning the proposed system relative to VI-based configurations. The added text is as follows:

A recent thermodynamic analysis has demonstrated that flexible heat pump cycles incorporating thermal energy storage (TES) can achieve COP improvements thermodynamically equivalent to two-stage and enhanced vapour injection (EVI) architectures, up to 24% depending on refrigerant selection, while retaining the simplicity of a standard single-stage compressor [22].

3. Refrigerant liquid through the four-way valve will significantly reduce switching reliability. This issue should be addressed in the system design.

Reply: We thank the reviewer for raising this important practical concern. We acknowledge that liquid refrigerant passing through a four-way valve during mode switching is a well-recognised reliability issue in reversible heat pump systems.

We wish to clarify that this concern has been directly addressed in the experimentally validated prototype of the underlying flexible heat pump system, reported in our previously published work [22]. In that implementation, a ball valve arrangement combined with check valves to control refrigerant flow direction during mode transitions. A liquid receiver is also incorporated to manage refrigerant charge during switching transients. Thermostatic expansion valves with feeler bulbs independently regulate superheat in each operating mode. This architecture was demonstrated experimentally to provide reliable and repeatable mode switching without liquid slugging.

The four-way valve representation in the present manuscript schematic was adopted for conceptual clarity. In practical implementation, the ball valve arrangement validated in [23] would be employed. A brief clarifying note has been added to the manuscript accordingly.

Mode switching reliability is ensured through a ball valve arrangement with integrated check valves and a liquid receiver, as validated experimentally in the prototype implementation of this system [23].

4. How will the system operate during summer?

Reply: We thank the reviewer for this practical question. During summer operation, two distinct operating strategies are feasible depending on ambient conditions and DHW demand:

In the baseline summer mode, when space heating demand is absent, the system operates as a conventional single-stage heat pump dedicated to DHW production, with the TES unit bypassed. This is a standard operating mode common to all heat pump systems serving dual SH and DHW functions.

However, the proposed system architecture also enables an advanced summer operating mode, in which the condenser (SH circuit) is bypassed and the TES unit serves as the primary heat sink. In this configuration, heat extracted from the warm ambient air via the evaporator is delivered directly to the TES during the charging phase. Given that summer ambient temperatures are substantially higher than the 7°C winter baseline, the temperature lift required to charge the TES to its target phase-change temperature is considerably reduced. The stored latent heat is subsequently discharged to produce DHW at 60–65°C. Whether this advanced mode outperforms direct single-stage DHW operation depends on the interplay between ambient temperature, DHW demand profile, and TES setpoint temperature — a trade-off that requires dynamic control optimisation to resolve.

A full dynamic control analysis comparing these summer operating strategies falls outside the scope of the present steady-state thermodynamic study but represents a valuable direction for future work, which the authors intend to pursue in subsequent investigations.

5. The analysis indicates that while HFO refrigerants offer higher energy efficiency, their low volumetric cooling capacity limits their practical applicability in this type of residential hot water system.

Reply: Thank you for this insightful comment. We agree that although HFO refrigerants (e.g., R1234yf and R1234ze) demonstrate higher energy efficiency, their relatively low volumetric heating capacity can limit their practical applicability in residential systems due to increased compressor size and system footprint.

To clarify this trade-off, we have added a discussion in the Results section highlighting the implications of volumetric capacity on system design. The added text is as follows:

While R1234yf and R1234ze demonstrate the highest power reduction potential, their relatively low volumetric heating capacity (VHC) must be considered for practical residential applications. Refrigerants with lower VHC require larger compressor displacement and piping to deliver the same heating capacity, which can increase system size and cost. Considering this trade-off, propane was selected as the representative refrigerant for detailed analysis in the preceding sections, as it provides a balanced combination of energy efficiency and volumetric capacity while also aligning with low-GWP requirements.

6. The manuscript text requires further proofreading. For example: The three-way valve is labeled G, not H.

Reply: Thank you for this comment. We have carefully proofread the manuscript and corrected this labelling error. Additional minor typographical issues have also been revised throughout the manuscript.

Reviewer #3 (Remarks to the Author):

In their paper "A quasi-two-stage heat pump for efficient dual-temperature supply - space heating and domestic hot water" submitted to Communications Engineering, the authors propose a new dual-mode heat pump system that integrates latent thermal energy storage inside

the unit to handle both space heating and domestic hot water more efficiently. In one mode it heats the space while charging the storage, and in the other it uses the stored heat to produce higher-temperature domestic hot water, reducing the need for electric backup heaters.

The authors develop a comprehensive quasi steady first law thermodynamic model to quantify the energy and power savings achievable with the proposed dual mode configuration. The model relies on: (i) a compressor efficiency that depends solely on the instantaneous pressure ratio; (ii) a fixed effectiveness for the evaporator unit; and (iii) a fixed pinch point temperature difference in the condenser (to the best of my understanding).

Using this framework, the paper evaluates system performance for various refrigerants and operating conditions. The results indicate that the proposed system can reduce electricity consumption by approximately 13 to 14% with a conventional baseline system, with R1234yf yielding the highest power reduction among the refrigerants considered. A key insight is that optimal control of the thermal energy storage temperature, as a function of both the DHW demand share and the space heating temperature, is crucial to maximizing the efficiency gains of the dual-mode operation.

Overall, the manuscript is well structured, the methodology is clearly laid out, and the results are convincingly presented and discussed. The topic is timely and relevant for residential energy systems, and the proposed concept has clear practical significance. I therefore recommend acceptance after minor revisions. My specific suggestions to further strengthen the manuscript are listed below.

1) Clarification and justification of modelling assumptions. The modelling assumptions and simplifications would benefit from a more detailed and transparent discussion. In particular, it is not entirely clear how the condenser is modelled in terms of exergy destruction (or heat exchanger effectiveness). Please clarify whether a specific heat exchanger model (e.g., epsilon-NTU, LMTD with a fixed pinch, or a simplified approach) is used to represent the condenser and how irreversibilities are accounted for. The assumption of a fixed evaporator effectiveness over the full range of operating conditions needs to be better justified. Since evaporator performance can vary with mass flow rate, air-side conditions, and refrigerant state, please discuss the implications of this simplification on the accuracy and robustness of the results (particularly important if several climate conditions are to be explores). If available, consider adding a brief sensitivity analysis (even qualitative) or referencing experimental/analytical studies that support the chosen effectiveness value.

Reply: Thank you for this insightful comment. We have clarified the heat exchanger modelling approach in the revised manuscript. Specifically, a simplified fixed-pinch temperature difference method is used to represent the condenser and evaporator, rather than detailed ε–NTU or LMTD formulations, with irreversibilities captured through finite temperature differences and pressure drop assumptions.

We have also provided further justification for the use of fixed evaporator effectiveness and clarified its implications for the comparative analysis.

The following text has been added to the manuscript as follows:

The condensers in both the SH and DHW loops are modeled using a fixed pinch temperature difference of 3.0°C. This approach ensures that the refrigerant condensation temperature is always sufficiently

higher than the required water delivery temperature (e.g., 48.0°C for 45.0°C SH) to facilitate effective heat transfer

And:

Regarding the evaporator, a fixed effectiveness of 0.57 is assumed for the baseline case study. While heat exchanger effectiveness in practice varies with mass flow rate and air-side conditions, a fixed value is utilized here to isolate the thermodynamic benefits of the DM-FHP cycle architecture from component-level transient variations. This value (0.57) was selected based on established performance data for residential air-source heat pumps operating under standard rating conditions (e.g., 7°C/3°C air inlet/outlet). Furthermore, because this study focuses on a comparative analysis between the DM-FHP and a standard ASHP, both systems are modeled with the same evaporator characteristics. Any fluctuations in effectiveness due to climate variations would affect both systems similarly, meaning the relative COP improvements reported remain a robust indicator of the cycle's superior architecture.

And:

Irreversibilities within the condensers are accounted for by a 5% pressure drop to represent realistic flow resistance and its subsequent impact on compressor work.

2) Influence of climate and ambient conditions. The current analysis appears to focus on a limited set of ambient air conditions. To better assess the value of the proposed concept, it would be beneficial to explore different climates. I encourage the authors to investigate the potential savings across a range of outdoor air temperatures (and, if feasible, humidities), representative of different climate zones. Even a parametric study using typical winter design temperatures for a few reference climates (e.g., mild, moderate, cold) would significantly strengthen the conclusions and highlight where the dual mode system provides the greatest benefit.

Reply: We thank the reviewer for this suggestion. Additional simulations were conducted at four ambient temperatures (0°C, 5°C, 7°C, and 10°C), and the results are presented in Figure R1 below.

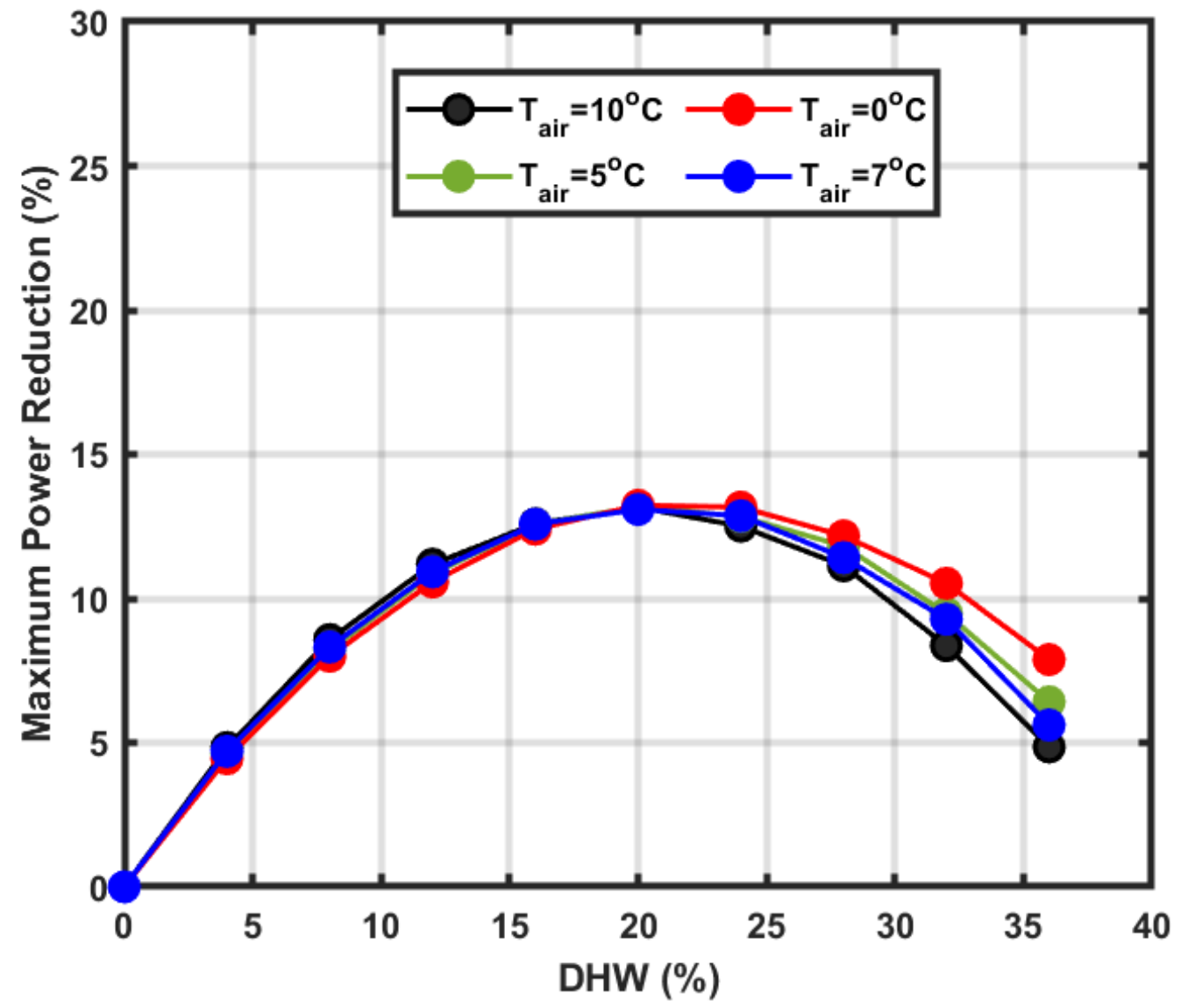

Figure. 1R. Maximum power reduction as a function of DHW share for propane at four ambient temperatures (0°C, 5°C, 7°C, and 10°C), at optimal TES temperature conditions.

As shown, as the DHW share increases, the influence of low ambient temperature becomes more pronounced on the maximum power reduction. This is because a larger portion of the operation shifts

from space heating, which is directly affected by ambient conditions, to DHW production, which is driven by TES discharge at a fixed and higher TES temperature. Unlike the standard system, where DHW production requires compressing refrigerant directly from the low-temperature evaporator and therefore incurs a disproportionately large performance penalty at cold ambient conditions, the flexible system operates the compressor between the fixed TES temperature and the DHW condensing temperature, entirely independent of outdoor air conditions.

Regarding humidity, while it primarily affects air-side heat transfer and frost formation on the evaporator, since the present study is a comparative analysis in which the same evaporator effectiveness ($\varepsilon = 0.57$) is applied consistently to both systems, the relative energy-saving trends remain robust across different humidity levels.

Beyond the scope of the present steady-state analysis, we intend to develop a dynamic simulation incorporating time-varying demand profiles and realistic ambient temperature across different climate zones, which will form the basis of a subsequent study.

3) Economic perspective: CAPEX vs OPEX. While the thermodynamic and electrical performance benefits are clearly demonstrated, the paper does not discuss the additional investment cost associated with implementing the dual mode heat pump and integrated latent thermal energy storage. The authors are strongly encouraged to provide at least indicative capital cost estimates for the proposed system relative to a standard single mode heat pump, even if these estimates come with wide uncertainty bands. A simple comparison of incremental CAPEX to expected OPEX savings (based on the reported electricity reductions and a reasonable electricity price) would greatly enhance the practical relevance of the work and help readers gauge the payback potential. If detailed cost data are unavailable, a qualitative discussion supported by literature-based cost ranges for thermal storage and additional components would still be very useful.

Reply: We thank the reviewer for this suggestion. As stated in the Methodology section, the present study adopts a series of thermodynamic assumptions to facilitate theoretical comparison between operating configurations. Consequently, a rigorous economic analysis, which requires detailed component sizing, real system costs, and realistic seasonal demand profiles, falls outside the defined scope of this work. As noted in the Conclusion section, future work will focus on coupled system simulations with realistic load profiles and control strategies, within which a comprehensive techno-economic assessment incorporating capital costs, and time-varying space heating and DHW demand profiles can be conducted.

With these minor revisions and clarifications, the manuscript will provide an even stronger and more comprehensive contribution to the literature on efficient residential heat pump systems for combined space heating and domestic hot water production.

Response to Reviewers
Manuscript Title: A quasi-two-stage heat pump for efficient dual-temperature supply – space heating and domestic hot water
Manuscript ID: COMMSENG-25-0770A
Authors: Zahra Hajabdollahi Ouderji, Muhammad Abid, Zhibin Yu

The requested revisions have been incorporated into the manuscript and are highlighted in yellow. These include the addition of a discussion on summer operation, the preparation of a Supplementary Information section containing Figure S1, revisions to the Figure 4 legend, and the formatting changes requested for figure files and captions.

In addition, the main figures have been prepared and uploaded as individual files, and all figure captions have been grouped at the end of the manuscript in accordance with the journal requirements.

Below, we provide a point-by-point response to the Editor's and Reviewers' comments.

We hope these revisions satisfactorily address all remaining comments and requirements. We thank the Editor and Reviewers again for their valuable guidance throughout the review process.

Sincerely,
The Authors

Point-by-point reply to the Editor' comments:

1- Please add a couple of sentences in the manuscript to explain the system operation during summer months, as mentioned by Referee 2.

Reply: Thank you, it was noted as below.

During summer, when space-heating demand is negligible or absent, both operating modes of the flexible heat pump, charging and discharging, can be employed for domestic hot water (DHW) production.

Because summer ambient temperatures are substantially higher than those encountered during winter operation, the temperature lift required for DHW production is significantly reduced in charging mode, where heat is upgraded directly from the ambient air to the DHW supply temperature of 60–65°C. Consequently, the charging mode operates more efficiently than in winter, a benefit that is also observed in conventional heat pumps.

However, the flexible heat pump offers an additional advantage. Thermal energy recovered and stored during charging mode can subsequently be utilised through discharging mode to provide DHW at 60–65°C. As in winter operation, the discharging mode benefits from a lower effective temperature lift and can therefore achieve a higher COP than a conventional heat pump producing DHW directly from the ambient air, even under summer conditions.

Consequently, the flexible heat pump is expected to maintain its performance advantage during summer operation. While both systems benefit from elevated ambient temperatures, the flexible heat pump can further exploit stored thermal energy through its high-efficiency discharging mode, resulting in improved overall efficiency and superior performance for DHW production.

2- Please add the simulation data provided in Figure R1 into a Supplementary Information section, and refer to this figure in the main manuscript.

Reply: Thank you, it was noted as below.

The influence of ambient air temperature on the maximum power reduction is further explored in Supplementary Figure S1, which presents results at four ambient temperatures (0°C, 5°C, 7°C, and 10°C) under optimal TES temperature conditions.
