# Supplementary material for: A quasi-two-stage heat pump for efficient dual—temperature supply—space heating and domestic hot water
Source: Commun Eng. 2026 Jul 21;5:194. doi: 10.1038/s44172-026-00739-9 (PMC13754852; doi:10.1038/s44172-026-00739-9)
Supplement: Supplementary file 2 [file 44172_2026_739_MOESM2_ESM.pdf]

Supplementary Information

As shown in Fig. S1, as the DHW share increases, the influence of low ambient temperature becomes more pronounced on the maximum power reduction. This is because a larger portion of the operation shifts from space heating, which is directly affected by ambient conditions, to DHW production, which is driven by TES discharge at a fixed and higher TES temperature. Unlike the standard system, where DHW production requires compressing refrigerant directly from the low-temperature evaporator and therefore incurs a disproportionately large performance penalty at cold ambient conditions, the flexible system operates the compressor between the fixed TES temperature and the DHW condensing temperature, entirely independent of outdoor air conditions.

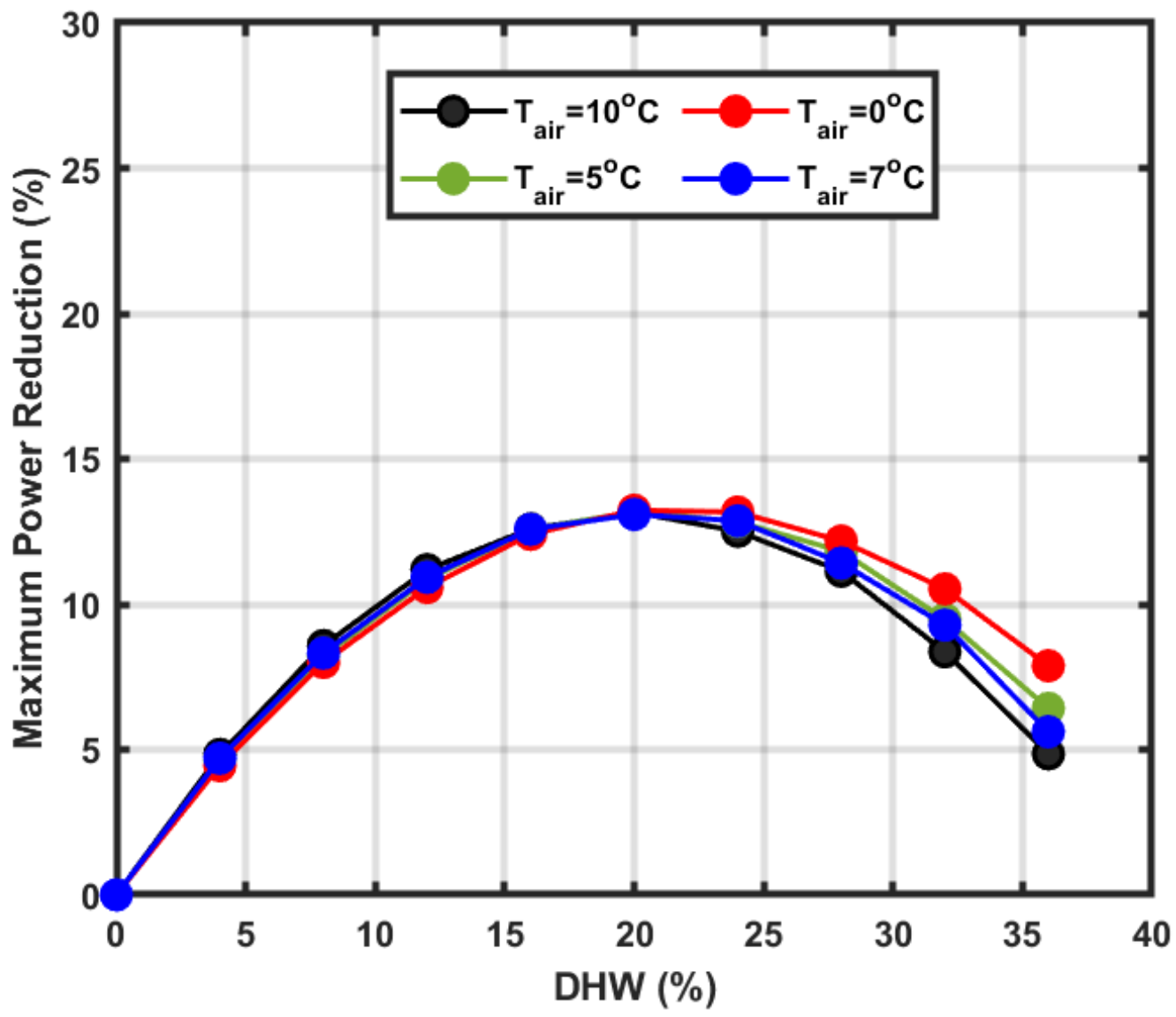

Figure S1. Maximum power reduction as a function of DHW share for propane at four ambient temperatures (0°C, 5°C, 7°C, and 10°C), at optimal TES temperature conditions.
